# Supplementary material for: Defining function of wild-type and three patient-specific TP53 mutations in a zebrafish model of embryonal rhabdomyosarcoma
Source: eLife. 2023 Jun 2;12:e68221. doi: 10.7554/eLife.68221 (PMC10322150; doi:10.7554/eLife.68221)
Supplement: Supplementary file 1. [file elife-68221-supp1.docx]

|  | *TP53^+/+^* | *TP53^153Δ^* | *TP53^C176F^* | *TP53^Y220C^* |
| --- | --- | --- | --- | --- |
| **Inhibits** *kRAS^G12D^/tp53^-/-^* mediated **ERMS tumor initiation?** | Yes (by 50%) | No change | YES (by 20%) | YES (by 20%) |
| **Promotes head localization** of *kRAS^G12D^/tp53^-/-^*ERMS tumors? | NO | YES | NO | YES |
| **Decreases tumor-cell proliferation** in *kRAS^G12D^/tp53^-/-^*? | YES | YES | YES | YES |
| **Increases tumor-cell apoptosis** in *kRAS^G12D^/tp53^-/-^* ? | YES | NO | YES | YES |
| **Has transactivation abilities** (*p21/cdkn1a*) | - | NO | NO | NO |
| **Has transactivation abilities** (*bbc3/puma*) | - | YES | YES | NO |
| **Has transactivation abilities** (*gadd45a*) | - | NO | NO | YES |
