# Supplementary material for: Defining function of wild-type and three patient-specific TP53 mutations in a zebrafish model of embryonal rhabdomyosarcoma
Source: eLife. 2023 Jun 2;12:e68221. doi: 10.7554/eLife.68221 (PMC10322150; doi:10.7554/eLife.68221)
Supplement: Supplementary file 2. [file elife-68221-supp2.docx]

**Supplementary Table 2.**

| **Symbol** | **baseMean P53LOF** | **baseMean TP53_P153** | **log2FC TP53_P153 over P53LOF** | **pval** | **padj** | **ENTREZID** | **GENENAME** |
| --- | --- | --- | --- | --- | --- | --- | --- |
| prdm8 | 3.7 | 9952.2 | 11.37 | 5.81E-44 | 7.48E-40 | 406719 | PR domain containing 8 |
| cldn19 | 2.2 | 7335.1 | 11.73 | 2.23E-43 | 8.06E-40 | 550431 | claudin 19 |
| nkx2.2a | 6.3 | 13052.0 | 11.02 | 2.26E-43 | 8.06E-40 | 30697 | NK2 homeobox 2a |
| six6b | 9.5 | 17777.1 | 10.86 | 2.50E-43 | 8.06E-40 | 553611 | SIX homeobox 6b |
| mag | 21.9 | 27693.0 | 10.31 | 6.08E-42 | 1.57E-38 | 474346 | myelin associated glycoprotein |
| sox10 | 15.6 | 20053.5 | 10.33 | 1.42E-41 | 3.04E-38 | 140616 | SRY-box transcription factor 10 |
| hepacama | 4.4 | 8313.6 | 10.90 | 2.10E-41 | 3.75E-38 | 100005952 | hepatic and glial cell adhesion molecule a |
| gpm6ab | 35.8 | 39053.5 | 10.09 | 2.57E-41 | 3.75E-38 | 407734 | glycoprotein M6Ab |
| plp1b | 60.7 | 63021.2 | 10.02 | 2.62E-41 | 3.75E-38 | 368234 | proteolipid protein 1b |
| cldnk | 19.9 | 22554.4 | 10.15 | 2.62E-40 | 3.37E-37 | 445070 | claudin k |
| etv1 | 1.7 | 4668.6 | 11.42 | 6.04E-39 | 7.07E-36 | 563876 | ETS variant transcription factor 1 |
| s100b | 28.8 | 20850.9 | 9.50 | 1.85E-37 | 1.98E-34 | 436825 | S100 calcium binding protein, beta (neural) |
| sema5a | 5.2 | 5043.9 | 9.92 | 2.35E-37 | 2.33E-34 | 561295 | sema domain, seven thrombospondin repeats (type 1 and type 1-like), transmembrane domain (TM) and short cytoplasmic domain, (semaphorin) 5A |
| zwi | 46.2 | 29633.7 | 9.33 | 5.70E-37 | 5.25E-34 | 336211 | zwilling |
| si:dkeyp-117h8.2 | 29.9 | 18995.9 | 9.31 | 2.13E-36 | 1.83E-33 | 570321 | si:dkeyp-117h8.2 |
| cd59 | 16.3 | 8888.4 | 9.09 | 6.33E-35 | 5.09E-32 | 567192 | CD59 molecule (CD59 blood group) |
| pou3f3b | 9.4 | 6310.1 | 9.40 | 7.01E-35 | 5.31E-32 | 30321 | POU class 3 homeobox 3b |
| crip2l | 3.6 | 3275.5 | 9.81 | 1.93E-34 | 1.38E-31 | 449795 | cysteine-rich protein 2-like |
| plp1a | 5.7 | 4257.9 | 9.54 | 2.77E-34 | 1.88E-31 | 64264 | proteolipid protein 1a |
| gadd45gb.1 | 1.4 | 2426.6 | 10.75 | 5.73E-34 | 3.69E-31 | 406507 | growth arrest and DNA-damage-inducible, gamma b, tandem duplicate 1 |
| fez1 | 9.3 | 4964.7 | 9.06 | 2.38E-33 | 1.46E-30 | 406705 | fasciculation and elongation protein zeta 1 (zygin I) |
| olig2 | 5.9 | 13971.2 | 11.21 | 6.33E-33 | 3.71E-30 | 325288 | oligodendrocyte lineage transcription factor 2 |
| flj13639 | 39.4 | 14426.5 | 8.52 | 8.83E-33 | 4.95E-30 | 677747 | flj13639 |
| mllt11 | 4.2 | 2749.9 | 9.36 | 9.87E-32 | 5.30E-29 | 563531 | MLLT11 transcription factor 7 cofactor |
| gng3 | 11.6 | 4356.3 | 8.55 | 1.32E-31 | 6.82E-29 | 114462 | guanine nucleotide binding protein (G protein), gamma 3 |
| pcbp4 | 2.0 | 1972.1 | 9.91 | 4.04E-31 | 2.00E-28 | 406391 | poly(rC) binding protein 4 |
| sox3 | 12.3 | 4269.2 | 8.43 | 5.66E-31 | 2.70E-28 | 30529 | SRY-box transcription factor 3 |
| zgc:113337 | 0.8 | 1478.8 | 10.91 | 2.98E-30 | 1.37E-27 | 503741 | zgc:113337 |
| pacsin1a | 10.3 | 7873.8 | 9.58 | 7.67E-30 | 3.41E-27 | 553559 | protein kinase C and casein kinase substrate in neurons 1a |
| sox2 | 20.8 | 14566.0 | 9.45 | 2.77E-29 | 1.19E-26 | 378723 | SRY-box transcription factor 2 |
| zgc:153426 | 5.3 | 2347.6 | 8.79 | 3.11E-29 | 1.29E-26 | 767746 | zgc:153426 |
| si:dkeyp-72h1.1 | 2.8 | 1799.9 | 9.32 | 3.97E-29 | 1.60E-26 | 799956 | si:dkeyp-72h1.1 |
| zgc:162989 | 4.6 | 2172.6 | 8.88 | 4.78E-29 | 1.87E-26 | 100037351 | zgc:162989 |
| fabp7a | 197.4 | 35717.4 | 7.50 | 5.20E-29 | 1.97E-26 | 58128 | fatty acid binding protein 7, brain, a |
| kif5aa | 3.0 | 1875.3 | 9.28 | 8.23E-29 | 3.03E-26 | 566086 | kinesin family member 5A, a |
| zgc:171558 | 0.4 | 1170.6 | 11.42 | 2.94E-28 | 1.05E-25 | 100141331 | zgc:171558 |
| amph | 2.4 | 1506.2 | 9.30 | 4.60E-28 | 1.60E-25 | 393804 | amphiphysin |
| stmn2a | 3.8 | 1606.6 | 8.73 | 3.80E-27 | 1.29E-24 | 449651 | stathmin 2a |
| nkx2.4b | 1.5 | 1274.1 | 9.78 | 4.75E-27 | 1.57E-24 | 58112 | NK2 homeobox 4b |
| cntd2 | 8.6 | 2306.5 | 8.06 | 5.09E-27 | 1.64E-24 | 561391 | cyclin N-terminal domain containing 2 |
| elmo2 | 31.6 | 5262.2 | 7.38 | 1.44E-26 | 4.51E-24 | 100006857 | engulfment and cell motility 2 |
| tmem125b | 0.6 | 1085.6 | 10.91 | 1.94E-26 | 5.95E-24 | 797385 | transmembrane protein 125b |
| dkk1a | 7.2 | 2083.2 | 8.18 | 2.84E-26 | 8.52E-24 | 799377 | dickkopf WNT signaling pathway inhibitor 1a |
| aplnra | 68.3 | 7765.2 | 6.83 | 4.03E-26 | 1.18E-23 | 561935 | apelin receptor a |
| zgc:172122 | 0.0 | 899.6 | Inf | 5.22E-26 | 1.49E-23 | 571844 | zgc:172122 |
| zgc:65894 | 29.4 | 4208.9 | 7.16 | 1.04E-25 | 2.92E-23 | 335798 | zgc:65894 |
| nmnat2 | 0.3 | 941.8 | 11.43 | 1.47E-25 | 4.04E-23 | 336257 | nicotinamide nucleotide adenylyltransferase 2 |
| slc1a2b | 78.6 | 25860.1 | 8.36 | 2.73E-25 | 7.32E-23 | 335836 | solute carrier family 1 member 2b |
| gfap | 21.3 | 15738.1 | 9.53 | 3.20E-25 | 8.40E-23 | 30646 | glial fibrillary acidic protein |
| elavl3 | 4.0 | 4120.3 | 10.01 | 8.89E-25 | 2.29E-22 | 30732 | ELAV like neuron-specific RNA binding protein 3 |
| pou3f3a | 24.7 | 2922.5 | 6.89 | 1.51E-24 | 3.81E-22 | 30386 | POU class 3 homeobox 3a |
| cdk5r2a | 0.3 | 849.6 | 11.28 | 3.24E-24 | 7.99E-22 | 550231 | cyclin-dependent kinase 5, regulatory subunit 2a (p39) |
| fyna | 96.9 | 9029.9 | 6.54 | 3.29E-24 | 7.99E-22 | 373872 | FYN proto-oncogene, Src family tyrosine kinase a |
| zgc:85722 | 13.6 | 2034.6 | 7.22 | 1.08E-23 | 2.56E-21 | 407982 | zgc:85722 |
| amer2 | 14.9 | 2065.2 | 7.12 | 4.07E-23 | 9.53E-21 | 100101649 | APC membrane recruitment protein 2 |
| syt11b | 3.0 | 1060.9 | 8.46 | 1.20E-22 | 2.77E-20 | 794707 | synaptotagmin XIb |
| atl1 | 14.7 | 1797.7 | 6.94 | 7.16E-22 | 1.62E-19 | 571909 | atlastin GTPase 1 |
| eno2 | 6.9 | 1264.3 | 7.51 | 1.33E-21 | 2.95E-19 | 402874 | enolase 2 |
| brinp1 | 0.0 | 609.7 | Inf | 2.58E-21 | 5.63E-19 | 100334375 | bone morphogenetic protein/retinoic acid inducible neural-specific 1 |
| brinp2 | 24.8 | 2374.4 | 6.58 | 2.80E-21 | 6.01E-19 | 561009 | bone morphogenetic protein/retinoic acid inducible neural-specific 2 |
| l1cama | 47.5 | 3105.0 | 6.03 | 3.39E-21 | 7.16E-19 | 30634 | L1 cell adhesion molecule, paralog a |
| sox9b | 357.1 | 23505.5 | 6.04 | 3.62E-21 | 7.52E-19 | 60642 | SRY-box transcription factor 9b |
| snap25a | 19.2 | 1988.4 | 6.70 | 4.31E-21 | 8.73E-19 | 30712 | synaptosome associated protein 25a |
| emp3b | 2.3 | 1567.1 | 9.44 | 4.41E-21 | 8.73E-19 | 560722 | epithelial membrane protein 3b |
| rab39bb | 2.0 | 973.2 | 8.94 | 4.43E-21 | 8.73E-19 | 566010 | RAB39B, member RAS oncogene family b |
| nlgn3a | 8.4 | 1961.0 | 7.86 | 4.48E-21 | 8.73E-19 | 562702 | neuroligin 3a |
| inka1a | 13.3 | 1534.0 | 6.85 | 9.39E-21 | 1.81E-18 | 692272 | inka box actin regulator 1a |
| wu:fj42b12 | 10.1 | 1396.5 | 7.11 | 1.04E-20 | 1.97E-18 | 100005187 | wu:fj42b12 |
| chga | 5.2 | 1045.3 | 7.65 | 5.80E-20 | 1.08E-17 | 450039 | chromogranin A |
| cntn5 | 2.5 | 755.1 | 8.25 | 7.25E-20 | 1.33E-17 | 327429 | contactin 5 |
| nexn | 4379.3 | 79.0 | -5.79 | 1.33E-19 | 2.42E-17 | 573301 | nexilin (F actin binding protein) |
| necap1 | 21.1 | 1698.5 | 6.33 | 1.46E-19 | 2.61E-17 | 393695 | NECAP endocytosis associated 1 |
| eef1a1a | 40.2 | 2721.3 | 6.08 | 1.72E-19 | 3.03E-17 | 336334 | eukaryotic translation elongation factor 1 alpha 1a |
| rnf144ab | 133.3 | 6209.0 | 5.54 | 2.88E-19 | 5.01E-17 | 437000 | ring finger protein 144ab |
| gria3a | 1.4 | 653.9 | 8.91 | 3.02E-19 | 5.18E-17 | 170452 | glutamate receptor, ionotropic, AMPA 3a |
| atp6v0a1b | 5.3 | 960.3 | 7.49 | 3.67E-19 | 6.22E-17 | 553691 | ATPase H+ transporting V0 subunit a1b |
| scdb | 82.6 | 7868.9 | 6.57 | 3.83E-19 | 6.40E-17 | 553734 | stearoyl-CoA desaturase b |
| dld | 29.1 | 1964.5 | 6.08 | 5.34E-19 | 8.81E-17 | 30138 | deltaD |
| olfm2a | 6.9 | 1227.8 | 7.48 | 7.13E-19 | 1.16E-16 | 334035 | olfactomedin 2a |
| gdap1 | 7.9 | 1033.7 | 7.03 | 9.94E-19 | 1.60E-16 | 553702 | ganglioside induced differentiation associated protein 1 |
| cdk5r1a | 0.0 | 464.4 | Inf | 1.71E-18 | 2.72E-16 | 568402 | cyclin-dependent kinase 5, regulatory subunit 1a (p35) |
| bcan | 101.7 | 22935.9 | 7.82 | 1.78E-18 | 2.80E-16 | 334113 | brevican |
| myog | 5982.7 | 130.1 | -5.52 | 1.90E-18 | 2.95E-16 | 30200 | myogenin |
| si:dkey-205h13.1 | 17.2 | 1328.7 | 6.28 | 3.15E-18 | 4.83E-16 | 559970 | si:dkey-205h13.1 |
| acbd7 | 39.1 | 2714.5 | 6.12 | 3.71E-18 | 5.63E-16 | 619256 | acyl-CoA binding domain containing 7 |
| bace1 | 66.1 | 3287.8 | 5.64 | 4.37E-18 | 6.55E-16 | 403005 | beta-secretase 1 |
| nsg2 | 4.4 | 779.1 | 7.47 | 5.00E-18 | 7.40E-16 | 324971 | neuronal vesicle trafficking associated 2 |
| ston2 | 379.6 | 14162.4 | 5.22 | 6.79E-18 | 9.94E-16 | 619262 | stonin 2 |
| tspan7b | 74.5 | 2873.4 | 5.27 | 1.46E-17 | 2.11E-15 | 449539 | tetraspanin 7b |
| gpm6aa | 2.9 | 653.1 | 7.81 | 1.53E-17 | 2.19E-15 | 368223 | glycoprotein M6Aa |
| pacsin1b | 17.3 | 1283.7 | 6.21 | 1.86E-17 | 2.63E-15 | 619246 | protein kinase C and casein kinase substrate in neurons 1b |
| cav3 | 1278.3 | 18.3 | -6.13 | 2.20E-17 | 3.08E-15 | 449679 | caveolin 3 |
| fsd1 | 10.6 | 995.2 | 6.55 | 2.80E-17 | 3.88E-15 | 566722 | fibronectin type III and SPRY domain containing 1 |
| tppp2 | 0.4 | 769.9 | 10.82 | 2.93E-17 | 4.01E-15 | 559490 | tubulin polymerization-promoting protein family member 2 |
| st8sia5 | 2.1 | 581.2 | 8.09 | 4.00E-17 | 5.42E-15 | 553754 | ST8 alpha-N-acetyl-neuraminide alpha-2,8-sialyltransferase 5 |
| spred3 | 4.8 | 1165.1 | 7.93 | 4.08E-17 | 5.48E-15 | 568925 | sprouty related EVH1 domain containing 3 |
| nrsn1 | 4.9 | 731.6 | 7.22 | 4.61E-17 | 6.12E-15 | 335551 | neurensin 1 |
| ugt8 | 3.8 | 676.6 | 7.49 | 5.03E-17 | 6.61E-15 | 553508 | UDP glycosyltransferase 8 |
| cdh4 | 22.8 | 1348.5 | 5.88 | 5.69E-17 | 7.40E-15 | 30297 | cadherin 4, type 1, R-cadherin (retinal) |
| vamp2 | 15.7 | 1329.4 | 6.40 | 6.11E-17 | 7.87E-15 | 336281 | vesicle-associated membrane protein 2 |
| tmem178b | 0.9 | 465.7 | 9.09 | 7.26E-17 | 9.26E-15 | 436669 | transmembrane protein 178B |
| cd247 | 975.4 | 12.3 | -6.31 | 8.46E-17 | 1.07E-14 | 100101654 | CD247 molecule |
| tlcd1 | 9.3 | 3047.7 | 8.36 | 9.77E-17 | 1.22E-14 | 450051 | TLC domain containing 1 |
| samd10a | 0.3 | 420.1 | 10.27 | 1.67E-16 | 2.07E-14 | 570533 | sterile alpha motif domain containing 10a |
| ctnnd2b | 35.2 | 2826.4 | 6.33 | 1.81E-16 | 2.23E-14 | 558069 | catenin (cadherin-associated protein), delta 2b |
| scg2b | 41.7 | 1742.5 | 5.39 | 1.86E-16 | 2.26E-14 | 777641 | secretogranin II (chromogranin C), b |
| epdr1 | 44.8 | 1877.6 | 5.39 | 1.90E-16 | 2.29E-14 | 436689 | ependymin related 1 |
| syn2a | 1.6 | 496.5 | 8.31 | 2.43E-16 | 2.90E-14 | 436870 | synapsin IIa |
| nrxn1a | 4.5 | 696.1 | 7.28 | 2.52E-16 | 2.97E-14 | 565531 | neurexin 1a |
| qki2 | 216.0 | 10927.2 | 5.66 | 2.86E-16 | 3.35E-14 | 393815 | QKI, KH domain containing, RNA binding 2 |
| evlb | 10.4 | 883.5 | 6.41 | 3.12E-16 | 3.62E-14 | 402929 | Enah/Vasp-like b |
| napgb | 33.4 | 1467.7 | 5.46 | 3.84E-16 | 4.41E-14 | 436742 | N-ethylmaleimide-sensitive factor attachment protein, gamma b |
| ntrk2a | 168.3 | 5960.3 | 5.15 | 4.54E-16 | 5.18E-14 | 65090 | neurotrophic tyrosine kinase, receptor, type 2a |
| kank1a | 117.1 | 4398.9 | 5.23 | 5.22E-16 | 5.89E-14 | 777729 | KN motif and ankyrin repeat domains 1a |
| klhl41b | 3822.1 | 116.8 | -5.03 | 6.01E-16 | 6.73E-14 | 321064 | kelch-like family member 41b |
| slmapb | 9.2 | 811.0 | 6.47 | 6.33E-16 | 7.03E-14 | 393146 | sarcolemma associated protein b |
| flot1a | 24.7 | 1299.7 | 5.72 | 9.03E-16 | 9.94E-14 | 561345 | flotillin 1a |
| pou3f2a | 3.2 | 573.4 | 7.50 | 1.01E-15 | 1.10E-13 | 30547 | POU class 3 homeobox 2a |
| actc1a | 2307.2 | 64.6 | -5.16 | 1.27E-15 | 1.38E-13 | 408256 | actin alpha cardiac muscle 1a |
| ascl1a | 1.5 | 473.9 | 8.33 | 1.51E-15 | 1.62E-13 | 30466 | achaete-scute family bHLH transcription factor 1a |
| opcml | 1.8 | 472.1 | 8.04 | 1.81E-15 | 1.92E-13 | 449538 | opioid binding protein/cell adhesion molecule-like |
| ptpro | 93.2 | 2781.2 | 4.90 | 1.82E-15 | 1.92E-13 | 407676 | protein tyrosine phosphatase receptor type O |
| syt11a | 93.6 | 3124.0 | 5.06 | 2.12E-15 | 2.22E-13 | 406769 | synaptotagmin XIa |
| itm2ca | 20.2 | 1101.6 | 5.77 | 2.46E-15 | 2.56E-13 | 436822 | integral membrane protein 2Ca |
| sult4a1 | 0.3 | 1195.5 | 11.78 | 3.47E-15 | 3.57E-13 | 678517 | sulfotransferase family 4A, member 1 |
| erbb3a | 7.6 | 3029.9 | 8.64 | 3.90E-15 | 3.96E-13 | 386635 | erb-b2 receptor tyrosine kinase 3a |
| cx27.5 | 6.6 | 656.1 | 6.64 | 3.90E-15 | 3.96E-13 | 114405 | connexin 27.5 |
| snai1a | 2739.2 | 91.3 | -4.91 | 4.00E-15 | 4.03E-13 | 30273 | snail family zinc finger 1a |
| mymk | 1085.0 | 5.2 | -7.71 | 4.14E-15 | 4.13E-13 | 415178 | myomaker, myoblast fusion factor |
| vax1 | 1.0 | 398.1 | 8.60 | 8.11E-15 | 8.04E-13 | 373870 | ventral anterior homeobox 1 |
| zgc:92518 | 1810.3 | 53.0 | -5.10 | 8.23E-15 | 8.09E-13 | 436634 | zgc:92518 |
| bcar3 | 69.6 | 2139.5 | 4.94 | 9.51E-15 | 9.28E-13 | 570610 | BCAR3 adaptor protein, NSP family member |
| phactr3a | 223.9 | 4588.0 | 4.36 | 1.02E-14 | 9.88E-13 | 556928 | phosphatase and actin regulator 3a |
| map3k12 | 1.0 | 394.5 | 8.68 | 1.06E-14 | 1.02E-12 | 404626 | mitogen-activated protein kinase kinase kinase 12 |
| trim109 | 840.6 | 13.8 | -5.93 | 1.27E-14 | 1.21E-12 | 723998 | tripartite motif containing 109 |
| hmga1b | 0.7 | 365.4 | 9.07 | 1.59E-14 | 1.51E-12 | 768130 | high mobility group AT-hook 1b |
| LOC110366352 | 24.7 | 1087.0 | 5.46 | 2.62E-14 | 2.47E-12 | 110366352 | uncharacterized LOC110366352 |
| tmem179aa | 29.1 | 1195.6 | 5.36 | 2.95E-14 | 2.75E-12 | 445310 | transmembrane protein 179a, genome duplicate A |
| dync1i1 | 45.8 | 1528.5 | 5.06 | 3.03E-14 | 2.80E-12 | 561525 | dynein, cytoplasmic 1, intermediate chain 1 |
| mapk10 | 1.4 | 387.9 | 8.15 | 4.59E-14 | 4.22E-12 | 569698 | mitogen-activated protein kinase 10 |
| six2a | 277.1 | 0.0 | -Inf | 4.74E-14 | 4.33E-12 | 83415 | SIX homeobox 2a |
| stmn1b | 104.4 | 11714.7 | 6.81 | 5.52E-14 | 5.01E-12 | 550548 | stathmin 1b |
| map4k2 | 10.1 | 708.7 | 6.13 | 6.56E-14 | 5.91E-12 | 678610 | mitogen-activated protein kinase kinase kinase kinase 2 |
| sox21b | 0.6 | 337.5 | 9.23 | 6.70E-14 | 5.95E-12 | 406246 | SRY-box transcription factor 21b |
| cited1 | 23.4 | 994.2 | 5.41 | 6.70E-14 | 5.95E-12 | 793303 | Cbp/p300-interacting transactivator, with Glu/Asp-rich carboxy-terminal domain, 1 |
| ncam1b | 22.2 | 1006.3 | 5.50 | 7.14E-14 | 6.30E-12 | 114442 | neural cell adhesion molecule 1b |
| npas1 | 16.6 | 921.4 | 5.79 | 8.12E-14 | 7.12E-12 | 559419 | neuronal PAS domain protein 1 |
| slc31a2 | 132.2 | 3236.0 | 4.61 | 8.35E-14 | 7.27E-12 | 563012 | solute carrier family 31 member 2 |
| rhoua | 22.7 | 965.6 | 5.41 | 9.31E-14 | 8.05E-12 | 492802 | ras homolog family member Ua |
| epb41l3a | 22.9 | 1784.6 | 6.29 | 9.74E-14 | 8.36E-12 | 445478 | erythrocyte membrane protein band 4.1-like 3a |
| si:dkey-33m11.6 | 1.3 | 369.8 | 8.15 | 1.17E-13 | 1.00E-11 | 555986 | si:dkey-33m11.6 |
| gulp1a | 109.8 | 2697.8 | 4.62 | 1.20E-13 | 1.01E-11 | 368404 | GULP PTB domain containing engulfment adaptor 1a |
| tlcd5a | 150.4 | 3587.9 | 4.58 | 1.24E-13 | 1.04E-11 | 562331 | TLC domain containing 5a |
| hes6 | 170.8 | 3820.6 | 4.48 | 1.30E-13 | 1.08E-11 | 373116 | hes family bHLH transcription factor 6 |
| opn5 | 0.0 | 270.4 | Inf | 1.43E-13 | 1.19E-11 | 564181 | opsin 5 |
| trim9 | 2.7 | 414.6 | 7.25 | 1.58E-13 | 1.30E-11 | 336099 | tripartite motif containing 9 |
| aqp9a | 2.4 | 2488.5 | 10.03 | 1.59E-13 | 1.31E-11 | 606660 | aquaporin 9a |
| tuba2 | 217.8 | 4430.8 | 4.35 | 1.88E-13 | 1.53E-11 | 406303 | tubulin, alpha 2 |
| fbxl16 | 6.6 | 554.2 | 6.40 | 2.16E-13 | 1.75E-11 | 797939 | F-box and leucine-rich repeat protein 16 |
| cntnap2b | 0.0 | 278.7 | Inf | 2.38E-13 | 1.92E-11 | 563345 | contactin associated protein like 2b |
| slc35g2a | 17.5 | 825.6 | 5.56 | 2.59E-13 | 2.07E-11 | 492818 | solute carrier family 35 member G2a |
| tnr | 24.8 | 25905.7 | 10.03 | 3.45E-13 | 2.74E-11 | 369191 | tenascin R (restrictin, janusin) |
| macc1 | 48.5 | 1453.2 | 4.90 | 3.90E-13 | 3.08E-11 | 558777 | MET transcriptional regulator MACC1 |
| fam219ab | 17.1 | 796.5 | 5.54 | 4.34E-13 | 3.41E-11 | 553801 | family with sequence similarity 219 member Ab |
| phyhiplb | 11.7 | 2056.9 | 7.46 | 5.94E-13 | 4.62E-11 | 100004913 | phytanoyl-CoA 2-hydroxylase interacting protein-like b |
| dscama | 4.5 | 3974.2 | 9.77 | 5.95E-13 | 4.62E-11 | 568643 | Down syndrome cell adhesion molecule a |
| zgc:172131 | 0.3 | 274.7 | 9.93 | 7.97E-13 | 6.15E-11 | 100002821 | zgc:172131 |
| cdk5r1b | 24.4 | 862.2 | 5.14 | 9.60E-13 | 7.36E-11 | 436788 | cyclin-dependent kinase 5, regulatory subunit 1b (p35) |
| serp2 | 2.5 | 377.0 | 7.24 | 1.00E-12 | 7.64E-11 | 100319240 | stress-associated endoplasmic reticulum protein family member 2 |
| dnm1b | 5.3 | 472.5 | 6.49 | 1.04E-12 | 7.85E-11 | 100333543 | dynamin 1b |
| syt1a | 68.4 | 1445.8 | 4.40 | 1.28E-12 | 9.67E-11 | 436736 | synaptotagmin Ia |
| il1rapl1b | 0.0 | 241.5 | Inf | 1.34E-12 | 1.00E-10 | 557801 | interleukin 1 receptor accessory protein-like 1b |
| cdkn2c | 197.3 | 3594.6 | 4.19 | 1.47E-12 | 1.10E-10 | 555797 | cyclin-dependent kinase inhibitor 2C (p18, inhibits CDK4) |
| pitx3 | 3120.1 | 163.2 | -4.26 | 1.63E-12 | 1.20E-10 | 402974 | paired-like homeodomain 3 |
| olfm1b | 9.6 | 589.6 | 5.94 | 1.63E-12 | 1.20E-10 | 445092 | olfactomedin 1b |
| soga3a | 38.1 | 1012.9 | 4.73 | 1.85E-12 | 1.36E-10 | 558933 | SOGA family member 3a |
| slc45a1 | 5.8 | 1022.2 | 7.47 | 2.05E-12 | 1.49E-10 | 791217 | solute carrier family 45 member 1 |
| mbpb | 1430.4 | 23408.3 | 4.03 | 2.23E-12 | 1.61E-10 | 368319 | myelin basic protein b |
| napba | 0.9 | 276.9 | 8.34 | 3.42E-12 | 2.46E-10 | 553682 | N-ethylmaleimide-sensitive factor attachment protein, beta a |
| csrp1b | 1.0 | 501.4 | 8.94 | 3.62E-12 | 2.59E-10 | 791132 | cysteine and glycine-rich protein 1b |
| rufy3 | 249.4 | 4316.6 | 4.11 | 3.84E-12 | 2.73E-10 | 541522 | RUN and FYVE domain containing 3 |
| ppdpfa | 5138.2 | 334.6 | -3.94 | 4.92E-12 | 3.48E-10 | 338304 | pancreatic progenitor cell differentiation and proliferation factor a |
| zgc:153383 | 15.8 | 659.5 | 5.38 | 5.34E-12 | 3.74E-10 | 751751 | zgc:153383 |
| meis2a | 1462.2 | 65.0 | -4.49 | 5.34E-12 | 3.74E-10 | 170454 | Meis homeobox 2a |
| foxd2 | 1039.6 | 32.5 | -5.00 | 6.18E-12 | 4.30E-10 | 30525 | forkhead box D2 |
| elovl7a | 0.3 | 246.2 | 9.77 | 6.79E-12 | 4.70E-10 | 334217 | ELOVL fatty acid elongase 7a |
| zgc:153981 | 51.6 | 1118.9 | 4.44 | 8.51E-12 | 5.86E-10 | 563986 | zgc:153981 |
| rtn1a | 517.5 | 8064.1 | 3.96 | 8.94E-12 | 6.11E-10 | 323706 | reticulon 1a |
| nova1 | 17.5 | 792.4 | 5.50 | 8.96E-12 | 6.11E-10 | 555590 | NOVA alternative splicing regulator 1 |
| mef2ca | 2000.0 | 109.1 | -4.20 | 1.06E-11 | 7.20E-10 | 30575 | myocyte enhancer factor 2ca |
| scn8aa | 2.9 | 536.1 | 7.53 | 1.52E-11 | 1.03E-09 | 58152 | sodium channel, voltage gated, type VIII, alpha subunit a |
| nptxrb | 4.0 | 373.1 | 6.54 | 1.64E-11 | 1.10E-09 | 100005257 | neuronal pentraxin receptor b |
| myh7ba | 435.4 | 6.6 | -6.05 | 1.86E-11 | 1.24E-09 | 100884147 | myosin, heavy chain 7B, cardiac muscle, beta a |
| tgm2b | 1328.2 | 68.2 | -4.28 | 2.31E-11 | 1.53E-09 | 323856 | transglutaminase 2b |
| cspg5b | 11.9 | 1628.8 | 7.09 | 2.54E-11 | 1.67E-09 | 559358 | chondroitin sulfate proteoglycan 5b |
| stac3 | 975.3 | 38.6 | -4.66 | 2.77E-11 | 1.81E-09 | 445111 | SH3 and cysteine rich domain 3 |
| hsp90aa1.1 | 19348.1 | 1365.0 | -3.83 | 2.77E-11 | 1.81E-09 | 30591 | heat shock protein 90, alpha (cytosolic), class A member 1, tandem duplicate 1 |
| acap3b | 12.7 | 1341.6 | 6.73 | 3.48E-11 | 2.27E-09 | 100009646 | ArfGAP with coiled-coil, ankyrin repeat and PH domains 3b |
| col5a2a | 16027.3 | 830.6 | -4.27 | 3.70E-11 | 2.39E-09 | 564821 | collagen, type V, alpha 2a |
| lnx2a | 0.0 | 192.8 | Inf | 4.62E-11 | 2.97E-09 | 553331 | ligand of numb-protein X 2a |
| vim | 157.6 | 13864.1 | 6.46 | 5.02E-11 | 3.21E-09 | 140599 | vimentin |
| rnd3b | 3411.6 | 234.2 | -3.86 | 5.03E-11 | 3.21E-09 | 436864 | Rho family GTPase 3b |
| myl9b | 2833.3 | 188.9 | -3.91 | 5.07E-11 | 3.22E-09 | 406493 | myosin, light chain 9b, regulatory |
| ccn1 | 2877.7 | 199.5 | -3.85 | 5.30E-11 | 3.34E-09 | 403033 | cellular communication network factor 1 |
| si:ch211-30b16.2 | 0.7 | 234.9 | 8.43 | 5.75E-11 | 3.61E-09 | 566679 | si:ch211-30b16.2 |
| cacna2d2b | 0.0 | 346.2 | Inf | 5.80E-11 | 3.63E-09 | 568759 | calcium channel, voltage-dependent, alpha 2/delta subunit 2b |
| ptgdsa | 0.0 | 192.9 | Inf | 6.32E-11 | 3.92E-09 | 555483 | prostaglandin D2 synthase a |
| kif3cb | 0.9 | 231.7 | 8.08 | 6.33E-11 | 3.92E-09 | 550547 | kinesin family member 3Cb |
| spire1a | 1.7 | 274.8 | 7.33 | 7.03E-11 | 4.33E-09 | 557962 | spire-type actin nucleation factor 1a |
| nrgna | 8.3 | 783.2 | 6.56 | 7.06E-11 | 4.33E-09 | 567608 | neurogranin (protein kinase C substrate, RC3) a |
| tnika | 82.8 | 1430.1 | 4.11 | 7.62E-11 | 4.65E-09 | 325042 | TRAF2 and NCK interacting kinase a |
| mdkb | 85.9 | 4542.6 | 5.72 | 8.02E-11 | 4.87E-09 | 65231 | midkine b |
| cntnap2a | 2.0 | 282.2 | 7.11 | 9.09E-11 | 5.50E-09 | 559157 | contactin associated protein like 2a |
| clcn4 | 106.6 | 1569.4 | 3.88 | 9.53E-11 | 5.74E-09 | 768175 | chloride channel, voltage-sensitive 4 |
| zgc:194578 | 1.4 | 256.6 | 7.56 | 9.65E-11 | 5.78E-09 | 792915 | zgc:194578 |
| si:ch211-241j12.3 | 10.7 | 494.7 | 5.53 | 1.04E-10 | 6.19E-09 | 566552 | si:ch211-241j12.3 |
| dpysl5a | 336.6 | 7088.9 | 4.40 | 1.09E-10 | 6.47E-09 | 324416 | dihydropyrimidinase like 5a |
| paqr7b | 31.5 | 891.7 | 4.82 | 1.09E-10 | 6.47E-09 | 368256 | progestin and adipoQ receptor family member VII, b |
| cdh11 | 152.4 | 4505.3 | 4.89 | 1.30E-10 | 7.64E-09 | 30461 | cadherin 11, type 2, OB-cadherin (osteoblast) |
| ppp3ca | 104.6 | 1713.6 | 4.03 | 1.46E-10 | 8.52E-09 | 794574 | protein phosphatase 3, catalytic subunit, alpha isozyme |
| ctsf | 789.6 | 9224.5 | 3.55 | 1.46E-10 | 8.52E-09 | 565588 | cathepsin F |
| cdk5r2b | 0.0 | 175.6 | Inf | 2.01E-10 | 1.17E-08 | 445114 | cyclin-dependent kinase 5, regulatory subunit 2b (p39) |
| lim2.2 | 27.6 | 3737.3 | 7.08 | 2.19E-10 | 1.27E-08 | 572341 | lens intrinsic membrane protein 2.2 |
| ywhag1 | 249.6 | 3315.6 | 3.73 | 2.57E-10 | 1.48E-08 | 117604 | 3-monooxygenase/tryptophan 5-monooxygenase activation protein, gamma polypeptide 1 |
| trpc1 | 7.1 | 408.0 | 5.85 | 2.62E-10 | 1.50E-08 | 570841 | transient receptor potential cation channel, subfamily C, member 1 |
| nsfa | 9.8 | 440.9 | 5.49 | 3.20E-10 | 1.82E-08 | 368483 | N-ethylmaleimide-sensitive factor a |
| zgc:114175 | 51.8 | 1014.9 | 4.29 | 3.24E-10 | 1.84E-08 | 619257 | zgc:114175 |
| naalad2 | 14.4 | 490.0 | 5.09 | 3.47E-10 | 1.96E-08 | 393247 | N-acetylated alpha-linked acidic dipeptidase 2 |
| tspan18b | 470.6 | 5275.7 | 3.49 | 3.57E-10 | 2.01E-08 | 436712 | tetraspanin 18b |
| ppp2r2ca | 14.1 | 498.4 | 5.14 | 3.62E-10 | 2.02E-08 | 100002802 | protein phosphatase 2, regulatory subunit B, gamma a |
| jupa | 903.3 | 42.6 | -4.40 | 3.63E-10 | 2.02E-08 | 30415 | junction plakoglobin a |
| pax7b | 477.7 | 13.4 | -5.16 | 3.90E-10 | 2.17E-08 | 566533 | paired box 7b |
| pbxip1b | 2356.7 | 128.8 | -4.19 | 4.08E-10 | 2.25E-08 | 553293 | pre-B-cell leukemia homeobox interacting protein 1b |
| syngr3b | 89.3 | 1386.8 | 3.96 | 4.10E-10 | 2.25E-08 | 570094 | synaptogyrin 3b |
| dvl2 | 771.1 | 34.3 | -4.49 | 4.10E-10 | 2.25E-08 | 323630 | dishevelled segment polarity protein 2 |
| si:ch211-81a5.8 | 385.8 | 7.1 | -5.76 | 4.26E-10 | 2.32E-08 | 560648 | si:ch211-81a5.8 |
| nrxn2b | 35.1 | 2278.3 | 6.02 | 4.29E-10 | 2.33E-08 | 560420 | neurexin 2b |
| serpinh1a | 1863.7 | 133.0 | -3.81 | 4.31E-10 | 2.33E-08 | 555328 | serpin peptidase inhibitor, clade H (heat shock protein 47), member 1a |
| si:ch211-113g11.6 | 1.0 | 221.4 | 7.80 | 4.41E-10 | 2.38E-08 | 559008 | si:ch211-113g11.6 |
| slc7a14a | 2.8 | 286.7 | 6.69 | 4.69E-10 | 2.52E-08 | 566270 | solute carrier family 7 member 14a |
| cdkl5 | 20.1 | 560.7 | 4.80 | 5.39E-10 | 2.88E-08 | 559341 | cyclin-dependent kinase-like 5 |
| cavin1b | 58.7 | 1467.4 | 4.64 | 5.73E-10 | 3.05E-08 | 561082 | caveolae associated protein 1b |
| mapk8ip1a | 18.2 | 541.7 | 4.90 | 6.42E-10 | 3.40E-08 | 100537717 | mitogen-activated protein kinase 8 interacting protein 1a |
| syt4 | 20.9 | 3763.7 | 7.50 | 6.64E-10 | 3.50E-08 | 335357 | synaptotagmin IV |
| lin28a | 0.0 | 159.6 | Inf | 7.37E-10 | 3.87E-08 | 394066 | lin-28 homolog A (C. elegans) |
| slc25a6 | 0.7 | 199.3 | 8.19 | 7.68E-10 | 4.02E-08 | 566370 | solute carrier family 25 member 6 |
| zgc:123035 | 1.4 | 225.1 | 7.37 | 8.68E-10 | 4.52E-08 | 777704 | zgc:123035 |
| apoeb | 2715.4 | 27408.2 | 3.34 | 1.03E-09 | 5.33E-08 | 30314 | apolipoprotein Eb |
| scn1ba | 6.8 | 325.9 | 5.58 | 1.07E-09 | 5.56E-08 | 555423 | sodium channel, voltage-gated, type I, beta a |
| sept8b | 86.6 | 2219.6 | 4.68 | 1.10E-09 | 5.67E-08 | 571702 | septin 8b |
| ass1 | 238.3 | 2705.6 | 3.50 | 1.17E-09 | 6.01E-08 | 447864 | argininosuccinate synthase 1 |
| rap2ab | 1.4 | 219.6 | 7.33 | 1.18E-09 | 6.03E-08 | 100003903 | RAP2A, member of RAS oncogene family b |
| abcc8 | 0.3 | 176.5 | 9.29 | 1.21E-09 | 6.17E-08 | 553281 | ATP-binding cassette, sub-family C (CFTR/MRP), member 8 |
| rgra | 0.0 | 347.8 | Inf | 1.26E-09 | 6.41E-08 | 550575 | retinal G protein coupled receptor a |
| inaa | 0.0 | 152.4 | Inf | 1.31E-09 | 6.62E-08 | 555251 | internexin neuronal intermediate filament protein, alpha a |
| znf516 | 176.3 | 1942.6 | 3.46 | 1.34E-09 | 6.76E-08 | 100003866 | zinc finger protein 516 |
| tmem266 | 14.7 | 473.3 | 5.01 | 1.50E-09 | 7.50E-08 | 791190 | transmembrane protein 266 |
| lrrc4.2 | 0.8 | 1095.1 | 10.48 | 1.56E-09 | 7.79E-08 | 566572 | leucine rich repeat containing 4.2 |
| caska | 638.9 | 6961.1 | 3.45 | 1.74E-09 | 8.64E-08 | 259195 | calcium/calmodulin-dependent serine protein kinase a |
| lipia | 884.6 | 43.0 | -4.36 | 1.92E-09 | 9.50E-08 | 445105 | lipase, member Ia |
| cd226 | 274.2 | 3150.2 | 3.52 | 2.10E-09 | 1.04E-07 | 791164 | CD226 molecule |
| rtn1b | 318.2 | 3293.6 | 3.37 | 2.11E-09 | 1.04E-07 | 327065 | reticulon 1b |
| cavin4a | 6771.9 | 155.1 | -5.45 | 2.44E-09 | 1.19E-07 | 552940 | caveolae associated protein 4a |
| vat1 | 222.0 | 2475.7 | 3.48 | 2.45E-09 | 1.19E-07 | 368752 | vesicle amine transport 1 |
| gpr176 | 1.6 | 206.5 | 6.99 | 2.82E-09 | 1.37E-07 | 561969 | G protein-coupled receptor 176 |
| klhl40a | 1443.0 | 30.4 | -5.57 | 2.97E-09 | 1.43E-07 | 553257 | kelch-like family member 40a |
| aimp1b | 1392.4 | 34.1 | -5.35 | 2.97E-09 | 1.43E-07 | 494049 | aminoacyl tRNA synthetase complex interacting multifunctional protein 1b |
| actn1 | 3174.9 | 300.6 | -3.40 | 2.98E-09 | 1.43E-07 | 560400 | actinin, alpha 1 |
| lrrc15 | 760.5 | 41.4 | -4.20 | 3.00E-09 | 1.43E-07 | 791200 | leucine rich repeat containing 15 |
| tspan2a | 63.6 | 1029.8 | 4.02 | 3.14E-09 | 1.50E-07 | 378854 | tetraspanin 2a |
| hoxc10a | 843.2 | 48.2 | -4.13 | 3.19E-09 | 1.51E-07 | 100535576 | homeobox C10a |
| brinp3a.1 | 11.5 | 417.0 | 5.18 | 3.34E-09 | 1.58E-07 | 569746 | bone morphogenetic protein/retinoic acid inducible neural-specific 3a, tandem duplicate 1 |
| tubb6 | 1474.2 | 115.8 | -3.67 | 3.59E-09 | 1.69E-07 | 550458 | tubulin, beta 6 class V |
| si:dkeyp-73d8.6 | 12.3 | 420.8 | 5.10 | 3.59E-09 | 1.69E-07 | 100170809 | si:dkeyp-73d8.6 |
| ndrg3a | 273.3 | 2816.7 | 3.37 | 3.69E-09 | 1.73E-07 | 30286 | ndrg family member 3a |
| stap2a | 53.0 | 856.7 | 4.02 | 3.77E-09 | 1.76E-07 | 327172 | signal transducing adaptor family member 2a |
| ptprub | 33.9 | 1464.8 | 5.43 | 3.80E-09 | 1.77E-07 | 335096 | protein tyrosine phosphatase receptor type Ub |
| si:ch73-380l3.2 | 4117.0 | 409.7 | -3.33 | 4.05E-09 | 1.88E-07 | 100003913 | si:ch73-380l3.2 |
| perp | 1804.1 | 154.9 | -3.54 | 4.13E-09 | 1.91E-07 | 494479 | p53 apoptosis effector related to pmp22 |
| clasp2 | 1857.2 | 18722.3 | 3.33 | 5.20E-09 | 2.39E-07 | 404604 | cytoplasmic linker associated protein 2 |
| padi2 | 33.9 | 1701.0 | 5.65 | 5.28E-09 | 2.42E-07 | 386792 | peptidyl arginine deiminase, type II |
| rab6bb | 28.5 | 622.3 | 4.45 | 5.57E-09 | 2.54E-07 | 541339 | RAB6B, member RAS oncogene family b |
| tmtops3b | 1.0 | 185.1 | 7.50 | 5.86E-09 | 2.67E-07 | 102031126 | teleost multiple tissue opsin 3b |
| vgll2a | 298.5 | 5.9 | -5.67 | 5.94E-09 | 2.69E-07 | 556856 | vestigial-like family member 2a |
| slc30a1b | 53.2 | 829.7 | 3.96 | 6.43E-09 | 2.91E-07 | 100038803 | solute carrier family 30 member 1b |
| fam219aa | 89.7 | 1755.8 | 4.29 | 6.60E-09 | 2.97E-07 | 445197 | family with sequence similarity 219 member Aa |
| gria4a | 15.1 | 1307.6 | 6.43 | 6.71E-09 | 3.01E-07 | 407735 | glutamate receptor, ionotropic, AMPA 4a |
| epyc | 1.3 | 193.7 | 7.18 | 7.10E-09 | 3.16E-07 | 550602 | epiphycan |
| 3-Sep | 21.0 | 518.2 | 4.63 | 7.33E-09 | 3.26E-07 | 554123 | septin 3 |
| sall3b | 91.3 | 8368.9 | 6.52 | 7.41E-09 | 3.28E-07 | 568041 | spalt-like transcription factor 3b |
| abl2 | 4215.9 | 401.2 | -3.39 | 7.87E-09 | 3.46E-07 | 570636 | c-abl oncogene 2, non-receptor tyrosine kinase |
| nfixb | 117.7 | 3699.9 | 4.97 | 8.83E-09 | 3.87E-07 | 678521 | nuclear factor I/Xb |
| nfixa | 48.7 | 2145.4 | 5.46 | 8.89E-09 | 3.87E-07 | 103909284 | nuclear factor I/Xa |
| mansc1 | 19.1 | 492.4 | 4.69 | 8.90E-09 | 3.87E-07 | 100009658 | MANSC domain containing 1 |
| capn2a | 925.4 | 65.2 | -3.83 | 9.19E-09 | 3.98E-07 | 541374 | calpain 2, (m/II) large subunit a |
| mkxa | 0.0 | 130.6 | Inf | 9.26E-09 | 4.00E-07 | 799136 | mohawk homeobox a |
| tgfb3 | 1290.7 | 88.1 | -3.87 | 9.58E-09 | 4.13E-07 | 369195 | transforming growth factor, beta 3 |
| syt9b | 44.1 | 15405.7 | 8.45 | 1.05E-08 | 4.50E-07 | 445476 | synaptotagmin IXb |
| ddc | 400.3 | 12.0 | -5.06 | 1.22E-08 | 5.20E-07 | 406651 | dopa decarboxylase |
| mtmr1b | 55.9 | 854.6 | 3.93 | 1.32E-08 | 5.59E-07 | 541517 | myotubularin related protein 1b |
| ascl1b | 39.5 | 1496.4 | 5.24 | 1.39E-08 | 5.86E-07 | 30478 | achaete-scute family bHLH transcription factor 1b |
| hsd17b12a | 38.6 | 650.7 | 4.07 | 1.41E-08 | 5.93E-07 | 327417 | hydroxysteroid (17-beta) dehydrogenase 12a |
| zgc:110796 | 2.0 | 218.1 | 6.78 | 1.41E-08 | 5.93E-07 | 503755 | zgc:110796 |
| helt | 0.3 | 199.3 | 9.47 | 1.51E-08 | 6.31E-07 | 404275 | helt bHLH transcription factor |
| sox1a | 0.0 | 122.3 | Inf | 1.71E-08 | 7.12E-07 | 436756 | SRY-box transcription factor 1a |
| dcbld1 | 59.4 | 2434.6 | 5.36 | 1.74E-08 | 7.25E-07 | 664747 | discoidin, CUB and LCCL domain containing 1 |
| sc:d189 | 1.5 | 311.3 | 7.75 | 1.94E-08 | 8.02E-07 | 100002334 | sc:d189 |
| zgc:194665 | 11.7 | 365.1 | 4.97 | 1.94E-08 | 8.03E-07 | 569012 | zgc:194665 |
| smpdl3b | 202.6 | 2.3 | -6.47 | 2.07E-08 | 8.51E-07 | 564392 | sphingomyelin phosphodiesterase acid like 3B |
| myt1la | 2.7 | 223.0 | 6.38 | 2.38E-08 | 9.78E-07 | 559505 | myelin transcription factor 1-like, a |
| cx47.1 | 0.0 | 119.7 | Inf | 2.43E-08 | 9.93E-07 | 447835 | connexin 47.1 |
| golga7ba | 52.4 | 739.6 | 3.82 | 2.47E-08 | 1.01E-06 | 550228 | golgin A7 family, member Ba |
| pdia8 | 5.7 | 290.2 | 5.68 | 2.53E-08 | 1.03E-06 | 445123 | protein disulfide isomerase family A, member 8 |
| kctd15b | 1.8 | 285.5 | 7.28 | 2.60E-08 | 1.05E-06 | 445133 | potassium channel tetramerization domain containing 15b |
| fbxl22 | 1055.9 | 29.3 | -5.17 | 2.81E-08 | 1.13E-06 | 503591 | F-box and leucine-rich repeat protein 22 |
| sypa | 0.0 | 262.0 | Inf | 3.08E-08 | 1.22E-06 | 559698 | synaptophysin a |
| il15 | 547.7 | 12.4 | -5.47 | 3.15E-08 | 1.25E-06 | 654826 | interleukin 15 |
| vclb | 1458.9 | 138.5 | -3.40 | 3.15E-08 | 1.25E-06 | 100334859 | vinculin b |
| fam222ba | 129.7 | 1340.2 | 3.37 | 3.29E-08 | 1.29E-06 | 561123 | family with sequence similarity 222 member Ba |
| brinp3a.2 | 9.6 | 1920.0 | 7.64 | 3.33E-08 | 1.30E-06 | 100001603 | bone morphogenetic protein/retinoic acid inducible neural-specific 3a, tandem duplicate 2 |
| tagln3a | 0.0 | 116.2 | Inf | 3.48E-08 | 1.36E-06 | 563096 | transgelin 3a |
| viml | 30.5 | 553.0 | 4.18 | 4.29E-08 | 1.65E-06 | 393746 | vimentin like |
| tmem182a | 567.4 | 11.0 | -5.68 | 4.29E-08 | 1.65E-06 | 503742 | transmembrane protein 182a |
| arf3a | 4.9 | 245.6 | 5.63 | 4.40E-08 | 1.69E-06 | 445047 | ADP-ribosylation factor 3a |
| ryr1b | 421.9 | 17.4 | -4.60 | 4.56E-08 | 1.74E-06 | 570245 | ryanodine receptor 1b (skeletal) |
| cox5b2 | 585.3 | 35.3 | -4.05 | 4.78E-08 | 1.82E-06 | 751638 | cytochrome c oxidase subunit 5B2 |
| pcdh17 | 25.1 | 513.1 | 4.36 | 5.05E-08 | 1.91E-06 | 561336 | protocadherin 17 |
| pkib | 1.9 | 185.7 | 6.63 | 5.34E-08 | 2.02E-06 | 797348 | protein kinase (cAMP-dependent, catalytic) inhibitor beta |
| tp53i11b | 281.4 | 3616.2 | 3.68 | 5.38E-08 | 2.02E-06 | 556618 | tumor protein p53 inducible protein 11b |
| lingo2b | 0.3 | 129.4 | 8.57 | 5.61E-08 | 2.09E-06 | 564052 | leucine rich repeat and Ig domain containing 2b |
| gatm | 1731.5 | 38.8 | -5.48 | 6.05E-08 | 2.25E-06 | 266799 | glycine amidinotransferase (L-arginine:glycine amidinotransferase) |
| si:dkeyp-73d8.9 | 28.8 | 1097.1 | 5.25 | 6.65E-08 | 2.46E-06 | 100170838 | si:dkeyp-73d8.9 |
| prkar1b | 26.7 | 495.2 | 4.21 | 6.70E-08 | 2.47E-06 | 767685 | protein kinase, cAMP-dependent, regulatory, type I, beta |
| hapln2 | 38.5 | 536.9 | 3.80 | 6.98E-08 | 2.57E-06 | 558291 | hyaluronan and proteoglycan link protein 2 |
| scpp8 | 658.2 | 44.6 | -3.88 | 7.03E-08 | 2.58E-06 | 100270761 | secretory calcium-binding phosphoprotein 8 |
| smyd2b | 755.7 | 54.4 | -3.80 | 7.06E-08 | 2.58E-06 | 568616 | SET and MYND domain containing 2b |
| slitrk6 | 5.0 | 246.1 | 5.61 | 7.90E-08 | 2.87E-06 | 324096 | SLIT and NTRK-like family, member 6 |
| dipk1b | 10.7 | 317.2 | 4.89 | 8.53E-08 | 3.09E-06 | 567731 | divergent protein kinase domain 1B |
| zgc:162952 | 54.0 | 745.9 | 3.79 | 8.67E-08 | 3.13E-06 | 555791 | zgc:162952 |
| gpr85 | 0.0 | 105.5 | Inf | 9.14E-08 | 3.27E-06 | 793299 | G protein-coupled receptor 85 |
| sb:cb470 | 3.4 | 1593.0 | 8.85 | 9.71E-08 | 3.46E-06 | 321270 | sb:cb470 |
| slc27a1b | 18.9 | 462.4 | 4.61 | 1.04E-07 | 3.68E-06 | 553512 | solute carrier family 27 member 1b |
| gpr173 | 0.0 | 104.6 | Inf | 1.09E-07 | 3.88E-06 | 57926 | G protein-coupled receptor 173 |
| ednrba | 3.5 | 206.7 | 5.88 | 1.25E-07 | 4.40E-06 | 30442 | endothelin receptor Ba |
| dpysl4 | 49.5 | 638.3 | 3.69 | 1.27E-07 | 4.47E-06 | 553411 | dihydropyrimidinase like 4 |
| chrng | 7120.5 | 131.6 | -5.76 | 1.29E-07 | 4.53E-06 | 100536659 | cholinergic receptor, nicotinic, gamma |
| cmya5 | 488.5 | 29.8 | -4.03 | 1.31E-07 | 4.57E-06 | 560687 | cardiomyopathy associated 5 |
| flrt2 | 77.9 | 815.2 | 3.39 | 1.42E-07 | 4.93E-06 | 571972 | fibronectin leucine rich transmembrane protein 2 |
| hip1rb | 23.2 | 445.9 | 4.26 | 1.47E-07 | 5.10E-06 | 562138 | huntingtin interacting protein 1 related b |
| hspb9 | 1726.6 | 72.1 | -4.58 | 1.63E-07 | 5.64E-06 | 100137108 | heat shock protein, alpha-crystallin-related, 9 |
| si:ch211-10a23.2 | 43.0 | 584.2 | 3.76 | 1.66E-07 | 5.75E-06 | 567812 | si:ch211-10a23.2 |
| chgb | 1.6 | 496.9 | 8.32 | 1.70E-07 | 5.85E-06 | 792853 | chromogranin B |
| slc12a5b | 0.0 | 118.9 | Inf | 1.85E-07 | 6.35E-06 | 797331 | solute carrier family 12 member 5b |
| stx1b | 5.7 | 237.2 | 5.37 | 1.87E-07 | 6.39E-06 | 58038 | syntaxin 1B |
| sox8b | 35.9 | 541.2 | 3.92 | 1.89E-07 | 6.42E-06 | 386704 | SRY-box transcription factor 8b |
| gria2b | 29.4 | 497.2 | 4.08 | 2.03E-07 | 6.89E-06 | 170451 | glutamate receptor, ionotropic, AMPA 2b |
| ptprb | 26.5 | 842.2 | 4.99 | 2.14E-07 | 7.24E-06 | 100149130 | protein tyrosine phosphatase receptor type b |
| sept4b | 30.2 | 470.4 | 3.96 | 2.18E-07 | 7.38E-06 | 100003446 | septin 4b |
| dpp6 | 48.0 | 630.5 | 3.72 | 2.25E-07 | 7.58E-06 | NA | NA |
| elovl4a | 35.1 | 556.0 | 3.99 | 2.33E-07 | 7.84E-06 | 393769 | ELOVL fatty acid elongase 4a |
| myod1 | 392.4 | 19.3 | -4.34 | 2.43E-07 | 8.16E-06 | 30513 | myogenic differentiation 1 |
| snap23.2 | 1436.3 | 40.7 | -5.14 | 2.54E-07 | 8.47E-06 | 641321 | synaptosome associated protein 23.2 |
| cnrip1a | 62.8 | 761.5 | 3.60 | 2.60E-07 | 8.64E-06 | 445213 | cannabinoid receptor interacting protein 1a |
| nupr1b | 779.6 | 69.9 | -3.48 | 2.60E-07 | 8.64E-06 | 799637 | nuclear protein 1b |
| slc6a9 | 41.7 | 593.9 | 3.83 | 2.72E-07 | 9.01E-06 | 494490 | solute carrier family 6 member 9 |
| vax2 | 0.0 | 153.8 | Inf | 3.04E-07 | 1.00E-05 | 373869 | ventral anterior homeobox 2 |
| lgals3b | 4988.8 | 434.4 | -3.52 | 3.04E-07 | 1.00E-05 | 325599 | lectin, galactoside binding soluble 3b |
| fndc3ba | 569.8 | 40.8 | -3.80 | 3.27E-07 | 1.07E-05 | 572313 | fibronectin type III domain containing 3Ba |
| cplx2l | 8.5 | 244.5 | 4.84 | 3.39E-07 | 1.11E-05 | 436732 | complexin 2, like |
| ppp1r1c | 2.0 | 156.8 | 6.30 | 3.57E-07 | 1.16E-05 | 436811 | protein phosphatase 1, regulatory (inhibitor) subunit 1C |
| pcsk1nl | 23.2 | 415.2 | 4.16 | 3.57E-07 | 1.16E-05 | 570305 | proprotein convertase subtilisin/kexin type 1 inhibitor, like |
| lin7b | 35.5 | 541.9 | 3.93 | 3.62E-07 | 1.17E-05 | 553768 | lin-7 homolog B (C. elegans) |
| mgll | 31.7 | 472.8 | 3.90 | 3.70E-07 | 1.20E-05 | 378960 | monoglyceride lipase |
| ctnna2 | 72.1 | 1740.1 | 4.59 | 3.74E-07 | 1.21E-05 | 567500 | catenin (cadherin-associated protein), alpha 2 |
| nxph1 | 15.5 | 1922.6 | 6.96 | 3.82E-07 | 1.23E-05 | 437006 | neurexophilin 1 |
| wdr17 | 4.2 | 189.9 | 5.50 | 4.13E-07 | 1.32E-05 | 561804 | WD repeat domain 17 |
| cyp2aa6 | 821.1 | 77.9 | -3.40 | 4.18E-07 | 1.33E-05 | 324212 | cytochrome P450, family 2, subfamily AA, polypeptide 6 |
| zgc:92429 | 1179.5 | 47.1 | -4.65 | 4.18E-07 | 1.33E-05 | 445063 | zgc:92429 |
| si:ch211-15e22.3 | 7.2 | 240.4 | 5.07 | 4.18E-07 | 1.33E-05 | 568915 | si:ch211-15e22.3 |
| ppp1r13ba | 3825.8 | 126.0 | -4.92 | 4.59E-07 | 1.46E-05 | 563577 | protein phosphatase 1, regulatory subunit 13Ba |
| xkr7 | 0.0 | 89.3 | Inf | 4.65E-07 | 1.47E-05 | 497073 | XK, Kell blood group complex subunit-related family, member 7 |
| gch2 | 10.3 | 474.7 | 5.53 | 4.76E-07 | 1.50E-05 | 64263 | GTP cyclohydrolase 2 |
| oxr1b | 27.6 | 825.9 | 4.91 | 4.96E-07 | 1.56E-05 | 570476 | oxidation resistance 1b |
| foxp3a | 475.0 | 32.7 | -3.86 | 5.04E-07 | 1.58E-05 | 571165 | forkhead box P3a |
| ifitm1 | 9635.4 | 703.7 | -3.78 | 5.11E-07 | 1.60E-05 | 100003209 | interferon induced transmembrane protein 1 |
| negr1 | 4.6 | 746.7 | 7.35 | 5.18E-07 | 1.61E-05 | 445374 | neuronal growth regulator 1 |
| sh2d3cb | 19.8 | 385.0 | 4.28 | 5.35E-07 | 1.66E-05 | 394051 | SH2 domain containing 3Cb |
| socs2 | 46.0 | 559.7 | 3.60 | 5.59E-07 | 1.73E-05 | 562062 | suppressor of cytokine signaling 2 |
| arrb1 | 0.3 | 103.8 | 8.25 | 5.60E-07 | 1.73E-05 | 553266 | arrestin, beta 1 |
| schip1 | 34.4 | 477.6 | 3.79 | 5.64E-07 | 1.74E-05 | 550605 | schwannomin interacting protein 1 |
| cdh7a | 1.0 | 156.9 | 7.35 | 5.94E-07 | 1.82E-05 | 567704 | cadherin 7a |
| ttc9b | 1.5 | 143.5 | 6.55 | 5.97E-07 | 1.82E-05 | 559732 | tetratricopeptide repeat domain 9B |
| zgc:194990 | 4.7 | 195.1 | 5.37 | 6.02E-07 | 1.83E-05 | 568410 | zgc:194990 |
| gphnb | 2.7 | 222.7 | 6.39 | 6.42E-07 | 1.95E-05 | 100007749 | gephyrin b |
| pdgfrl | 107.6 | 0.7 | -7.27 | 6.48E-07 | 1.96E-05 | 393284 | platelet-derived growth factor receptor-like |
| six2b | 1919.7 | 32.4 | -5.89 | 6.55E-07 | 1.98E-05 | 566454 | SIX homeobox 2b |
| cyp2ad6 | 181.0 | 4.1 | -5.45 | 6.94E-07 | 2.08E-05 | 799957 | cytochrome P450, family 2, subfamily AD, polypeptide 6 |
| yap1 | 246.6 | 8.7 | -4.82 | 7.02E-07 | 2.10E-05 | 561411 | Yes-associated protein 1 |
| rtn4r | 2.4 | 156.3 | 6.03 | 7.42E-07 | 2.22E-05 | 403306 | reticulon 4 receptor |
| gnb5a | 11.4 | 362.6 | 4.99 | 7.59E-07 | 2.26E-05 | 562813 | guanine nucleotide binding protein (G protein), beta 5a |
| map7d2a | 52.0 | 611.6 | 3.56 | 8.64E-07 | 2.56E-05 | 100005195 | MAP7 domain containing 2a |
| nkain2 | 10.7 | 285.2 | 4.74 | 8.75E-07 | 2.58E-05 | 110438188 | sodium/potassium transporting ATPase interacting 2 |
| zgc:158689 | 10.9 | 1554.5 | 7.16 | 9.06E-07 | 2.67E-05 | 791177 | zgc:158689 |
| dcc | 0.0 | 82.0 | Inf | 9.76E-07 | 2.86E-05 | 569360 | DCC netrin 1 receptor |
| olfm1a | 2.4 | 899.8 | 8.56 | 1.01E-06 | 2.96E-05 | 436764 | olfactomedin 1a |
| nr0b1 | 15.3 | 393.1 | 4.69 | 1.02E-06 | 2.99E-05 | 100001692 | nuclear receptor subfamily 0, group B, member 1 |
| egfra | 250.9 | 2925.7 | 3.54 | 1.12E-06 | 3.22E-05 | 378478 | epidermal growth factor receptor a (erythroblastic leukemia viral (v-erb-b) oncogene homolog, avian) |
| pbx3b | 7.9 | 244.8 | 4.95 | 1.12E-06 | 3.23E-05 | 58140 | pre-B-cell leukemia homeobox 3b |
| mir133b | 78.2 | 0.0 | -Inf | 1.17E-06 | 3.35E-05 | 100033654 | microRNA 133b |
| hey1 | 1350.4 | 104.2 | -3.70 | 1.26E-06 | 3.58E-05 | 58008 | hes-related family bHLH transcription factor with YRPW motif 1 |
| actl6b | 0.9 | 141.4 | 7.37 | 1.31E-06 | 3.71E-05 | 553775 | actin-like 6B |
| nppc | 1.8 | 133.1 | 6.17 | 1.31E-06 | 3.71E-05 | 570244 | natriuretic peptide C |
| ephb4b | 700.3 | 68.6 | -3.35 | 1.32E-06 | 3.73E-05 | 30691 | eph receptor B4b |
| sdcbp | 19.7 | 342.0 | 4.12 | 1.50E-06 | 4.20E-05 | 407719 | syndecan binding protein (syntenin) |
| ccdc28b | 2.6 | 151.8 | 5.85 | 1.53E-06 | 4.29E-05 | 559814 | coiled-coil domain containing 28B |
| s1pr5a | 744.3 | 38.6 | -4.27 | 1.73E-06 | 4.80E-05 | 492350 | sphingosine-1-phosphate receptor 5a |
| hoxa3a | 426.4 | 30.2 | -3.82 | 1.86E-06 | 5.15E-05 | 58049 | homeobox A3a |
| stmn2b | 0.4 | 91.2 | 7.74 | 1.92E-06 | 5.30E-05 | 100003563 | stathmin 2b |
| cadm2b | 2.3 | 170.5 | 6.24 | 2.20E-06 | 6.03E-05 | 571698 | cell adhesion molecule 2b |
| gpr27 | 5.9 | 323.3 | 5.79 | 2.33E-06 | 6.37E-05 | 567355 | G protein-coupled receptor 27 |
| npdc1a | 29.1 | 391.3 | 3.75 | 2.35E-06 | 6.42E-05 | 794093 | neural proliferation, differentiation and control, 1a |
| dnajc5aa | 10.0 | 242.2 | 4.60 | 2.46E-06 | 6.68E-05 | 386768 | DnaJ (Hsp40) homolog, subfamily C, member 5aa |
| sgcd | 363.6 | 23.1 | -3.98 | 2.48E-06 | 6.70E-05 | 324961 | sarcoglycan, delta (dystrophin-associated glycoprotein) |
| cxcl18b | 9006.9 | 782.5 | -3.52 | 2.55E-06 | 6.89E-05 | 795785 | chemokine (C-X-C motif) ligand 18b |
| angpt2b | 11.5 | 269.4 | 4.55 | 2.59E-06 | 6.97E-05 | 114408 | angiopoietin 2b |
| nbeaa | 17.5 | 754.5 | 5.43 | 2.72E-06 | 7.31E-05 | 541373 | neurobeachin a |
| rpe65a | 3.0 | 146.4 | 5.61 | 2.76E-06 | 7.38E-05 | 393724 | retinoid isomerohydrolase RPE65 a |
| bnc2 | 341.2 | 23.5 | -3.86 | 2.79E-06 | 7.43E-05 | 559327 | basonuclin 2 |
| lhfpl3 | 4.8 | 179.7 | 5.21 | 2.88E-06 | 7.64E-05 | 445050 | LHFPL tetraspan subfamily member 3 |
| fnbp1a | 0.9 | 107.1 | 6.89 | 2.96E-06 | 7.86E-05 | 558990 | formin binding protein 1a |
| hoxc8a | 432.0 | 25.3 | -4.09 | 3.08E-06 | 8.13E-05 | 449648 | homeobox C8a |
| casq1b | 910.3 | 15.4 | -5.89 | 3.47E-06 | 9.16E-05 | 767757 | calsequestrin 1b |
| si:ch211-147k10.6 | 0.0 | 69.6 | Inf | 3.49E-06 | 9.20E-05 | 497538 | si:ch211-147k10.6 |
| eve1 | 133.9 | 2.4 | -5.78 | 3.68E-06 | 9.68E-05 | 30335 | even-skipped-like1 |
| ccndx | 2.3 | 734.1 | 8.30 | 3.82E-06 | 0.00010 | 567411 | cyclin Dx |
| kcnk3a | 0.0 | 69.0 | Inf | 4.02E-06 | 0.00010 | 561866 | potassium channel, subfamily K, member 3a |
| mturn | 0.0 | 68.4 | Inf | 4.03E-06 | 0.00010 | 792465 | maturin, neural progenitor differentiation regulator homolog (Xenopus) |
| slc38a5b | 2664.3 | 262.8 | -3.34 | 4.27E-06 | 0.00011 | 436921 | solute carrier family 38 member 5b |
| hspa1b | 341.1 | 24.0 | -3.83 | 4.29E-06 | 0.00011 | 798846 | heat shock protein family A (Hsp70) member 1B |
| ccdc85a | 0.8 | 93.1 | 6.92 | 4.39E-06 | 0.00011 | 100141348 | coiled-coil domain containing 85A |
| six4a | 480.2 | 19.0 | -4.66 | 4.90E-06 | 0.00012 | 65233 | SIX homeobox 4a |
| ksr2 | 4.3 | 204.1 | 5.55 | 4.90E-06 | 0.00012 | 569127 | kinase suppressor of ras 2 |
| ank2b | 104.4 | 2455.3 | 4.56 | 4.99E-06 | 0.00013 | 450043 | ankyrin 2b, neuronal |
| si:dkey-174m14.3 | 20.2 | 1475.8 | 6.19 | 5.01E-06 | 0.00013 | 563117 | si:dkey-174m14.3 |
| insyn1 | 1.1 | 183.9 | 7.35 | 5.07E-06 | 0.00013 | 100003007 | inhibitory synaptic factor 1 |
| csrp1a | 3740.9 | 290.3 | -3.69 | 5.60E-06 | 0.00014 | 378726 | cysteine and glycine-rich protein 1a |
| fzd3a | 3.4 | 143.9 | 5.40 | 5.64E-06 | 0.00014 | 565921 | frizzled class receptor 3a |
| sybu | 10.0 | 407.0 | 5.35 | 5.76E-06 | 0.00014 | 568207 | syntabulin (syntaxin-interacting) |
| tbx18 | 161.4 | 5.0 | -5.00 | 5.88E-06 | 0.00015 | 572713 | T-box transcription factor 18 |
| im:7152348 | 389.3 | 33.2 | -3.55 | 5.90E-06 | 0.00015 | 559250 | im:7152348 |
| cdh10a | 2.4 | 120.6 | 5.66 | 5.99E-06 | 0.00015 | 568370 | cadherin 10, type 2a (T2-cadherin) |
| b3gnt2a | 0.6 | 89.1 | 7.31 | 6.14E-06 | 0.00015 | 405766 | UDP-GlcNAc:betaGal beta-1,3-N-acetylglucosaminyltransferase 2a |
| trim101 | 552.8 | 18.8 | -4.87 | 6.19E-06 | 0.00015 | 445187 | tripartite motif containing 101 |
| col9a3 | 19.2 | 2071.9 | 6.76 | 6.20E-06 | 0.00015 | 567110 | collagen, type IX, alpha 3 |
| osbpl3a | 284.6 | 19.6 | -3.86 | 6.26E-06 | 0.00015 | 447908 | oxysterol binding protein-like 3a |
| scn3b | 56.6 | 650.0 | 3.52 | 6.46E-06 | 0.00016 | 563935 | sodium channel, voltage-gated, type III, beta |
| fa2h | 24.5 | 335.7 | 3.78 | 6.49E-06 | 0.00016 | 792257 | fatty acid 2-hydroxylase |
| gabrg2 | 0.0 | 103.3 | Inf | 6.52E-06 | 0.00016 | 553402 | gamma-aminobutyric acid (GABA) A receptor, gamma 2 |
| dpysl2b | 52.0 | 738.2 | 3.83 | 6.53E-06 | 0.00016 | 553412 | dihydropyrimidinase like 2b |
| znf219 | 13.4 | 241.8 | 4.18 | 6.74E-06 | 0.00016 | 102997060 | zinc finger protein 219 |
| clvs2 | 1.0 | 98.0 | 6.58 | 6.83E-06 | 0.00017 | 566769 | clavesin 2 |
| asic1a | 2.6 | 380.8 | 7.22 | 7.01E-06 | 0.00017 | 791696 | acid-sensing (proton-gated) ion channel 1a |
| c25h12orf75 | 2.1 | 471.7 | 7.79 | 7.76E-06 | 0.00018 | 100535414 | chromosome 25 C12orf75 homolog |
| cdhr1a | 3.0 | 141.7 | 5.57 | 8.44E-06 | 0.00020 | 449008 | cadherin-related family member 1a |
| rab15 | 4.7 | 165.6 | 5.15 | 8.51E-06 | 0.00020 | 436590 | RAB15, member RAS oncogene family |
| nkx6.2 | 0.3 | 243.5 | 9.76 | 8.77E-06 | 0.00021 | 565782 | NK6 homeobox 2 |
| popdc2 | 143.7 | 3.7 | -5.29 | 8.86E-06 | 0.00021 | 793666 | popeye domain containing 2 |
| LOC405768 | 228.9 | 4.8 | -5.59 | 9.25E-06 | 0.00022 | 405768 | protein kinase Npk |
| slc43a1b | 618.0 | 14.3 | -5.44 | 9.84E-06 | 0.00023 | 100009631 | solute carrier family 43 member 1b |
| stmn4 | 2.9 | 130.8 | 5.49 | 1.03E-05 | 0.00024 | 406710 | stathmin-like 4 |
| zgc:172145 | 409.3 | 37.8 | -3.44 | 1.06E-05 | 0.00024 | 559095 | zgc:172145 |
| slc22a2 | 459.9 | 45.8 | -3.33 | 1.14E-05 | 0.00026 | 406424 | solute carrier family 22 member 2 |
| gli3 | 324.4 | 25.0 | -3.70 | 1.22E-05 | 0.00028 | 403042 | GLI family zinc finger 3 |
| lsamp | 4.5 | 246.4 | 5.79 | 1.24E-05 | 0.00028 | 664692 | limbic system associated membrane protein |
| csnk1e | 14.6 | 247.4 | 4.09 | 1.25E-05 | 0.00028 | 100006858 | casein kinase 1, epsilon |
| slco5a1b | 11.2 | 892.7 | 6.32 | 1.34E-05 | 0.00030 | 101883455 | solute carrier organic anion transporter family member 5A1b |
| si:ch211-131k2.3 | 906.0 | 36.7 | -4.63 | 1.36E-05 | 0.00031 | 566751 | si:ch211-131k2.3 |
| olig1 | 0.0 | 55.9 | Inf | 1.52E-05 | 0.00034 | 100001484 | oligodendrocyte transcription factor 1 |
| syn3 | 3.1 | 123.6 | 5.33 | 1.57E-05 | 0.00035 | 107980438 | synapsin III |
| si:dkey-70p6.1 | 0.0 | 233.4 | Inf | 1.59E-05 | 0.00035 | 570575 | si:dkey-70p6.1 |
| anxa13l | 38.4 | 499.3 | 3.70 | 1.85E-05 | 0.00041 | 554118 | annexin A13, like |
| LOC103910026 | 1445.6 | 64.6 | -4.48 | 1.86E-05 | 0.00041 | 103910026 | PDZ and LIM domain protein 7-like |
| b4galt2 | 2.7 | 202.1 | 6.25 | 1.90E-05 | 0.00042 | 100148828 | UDP-Gal:betaGlcNAc beta 1,4- galactosyltransferase, polypeptide 2 |
| scn1lab | 28.8 | 1255.8 | 5.44 | 1.97E-05 | 0.00043 | 559447 | sodium channel, voltage-gated, type I like, alpha b |
| bhmt | 10107.4 | 588.1 | -4.10 | 1.98E-05 | 0.00043 | 322228 | betaine-homocysteine methyltransferase |
| jph3 | 5.5 | 152.3 | 4.80 | 2.01E-05 | 0.00044 | 569861 | junctophilin 3 |
| smyd1b | 3107.0 | 133.2 | -4.54 | 2.16E-05 | 0.00046 | 569027 | SET and MYND domain containing 1b |
| mid1ip1a | 380.0 | 12.3 | -4.95 | 2.17E-05 | 0.00047 | 30600 | MID1 interacting protein 1a |
| usp54a | 21.6 | 1275.3 | 5.88 | 2.20E-05 | 0.00047 | 563912 | ubiquitin specific peptidase 54a |
| nol4lb | 52.0 | 804.7 | 3.95 | 2.46E-05 | 0.00052 | 791170 | nucleolar protein 4-like b |
| mafaa | 239.5 | 4.7 | -5.67 | 2.62E-05 | 0.00055 | 100000492 | v-maf avian musculoaponeurotic fibrosarcoma oncogene homolog Aa |
| chst3a | 6.1 | 720.3 | 6.87 | 2.65E-05 | 0.00056 | 559721 | carbohydrate (chondroitin 6) sulfotransferase 3a |
| sema3fa | 3.3 | 152.6 | 5.53 | 2.78E-05 | 0.00059 | 544658 | sema domain, immunoglobulin domain (Ig), short basic domain, secreted, (semaphorin) 3Fa |
| tyro3 | 31.1 | 321.6 | 3.37 | 3.05E-05 | 0.00064 | 30709 | TYRO3 protein tyrosine kinase |
| cadm3 | 32.2 | 361.8 | 3.49 | 3.11E-05 | 0.00065 | 692273 | cell adhesion molecule 3 |
| rab3ab | 0.7 | 72.8 | 6.74 | 3.11E-05 | 0.00065 | 550457 | RAB3A, member RAS oncogene family, b |
| lin7a | 11.8 | 194.8 | 4.05 | 3.29E-05 | 0.00068 | 393682 | lin-7 homolog A (C. elegans) |
| cyp39a1 | 6.6 | 149.1 | 4.50 | 3.33E-05 | 0.00069 | 564475 | cytochrome P450, family 39, subfamily A, polypeptide 1 |
| samsn1b | 415.0 | 28.4 | -3.87 | 3.43E-05 | 0.00071 | 450018 | SAM domain, SH3 domain and nuclear localisation signals 1b |
| vmo1a | 219.4 | 14.4 | -3.93 | 3.44E-05 | 0.00071 | 793369 | vitelline membrane outer layer 1 homolog a |
| si:ch211-193l2.6 | 1.1 | 85.4 | 6.24 | 3.55E-05 | 0.00073 | 561947 | si:ch211-193l2.6 |
| zgc:153704 | 0.0 | 603.8 | Inf | 3.65E-05 | 0.00075 | 767759 | zgc:153704 |
| si:ch73-233f7.1 | 3.6 | 112.0 | 4.96 | 3.65E-05 | 0.00075 | 100537710 | si:ch73-233f7.1 |
| pip5k1cb | 0.0 | 72.5 | Inf | 3.76E-05 | 0.00077 | 100005033 | phosphatidylinositol-4-phosphate 5-kinase, type I, gamma b |
| lims2 | 139.8 | 1.4 | -6.69 | 3.82E-05 | 0.00078 | 553696 | LIM and senescent cell antigen-like domains 2 |
| dsc2l | 239.2 | 18.4 | -3.70 | 3.83E-05 | 0.00078 | 560091 | desmocollin 2 like |
| gli2b | 363.2 | 20.9 | -4.12 | 3.87E-05 | 0.00079 | 548610 | GLI family zinc finger 2b |
| zgc:194629 | 0.0 | 276.7 | Inf | 4.26E-05 | 0.00086 | 100004339 | zgc:194629 |
| slc8a2b | 4.8 | 163.0 | 5.08 | 4.27E-05 | 0.00086 | 567212 | solute carrier family 8 member 2b |
| nkx2.4a | 1.9 | 1224.0 | 9.36 | 4.40E-05 | 0.00088 | 562300 | NK2 homeobox 4a |
| scn2b | 11.2 | 197.6 | 4.14 | 4.43E-05 | 0.00088 | 777666 | sodium channel, voltage-gated, type II, beta |
| trim46b | 2.7 | 104.0 | 5.28 | 4.56E-05 | 0.00090 | 100034631 | tripartite motif containing 46b |
| hoxa9a | 200.6 | 12.8 | -3.97 | 4.85E-05 | 0.00096 | 58047 | homeobox A9a |
| gap43 | 10.3 | 738.9 | 6.17 | 5.15E-05 | 0.0010 | 30608 | growth associated protein 43 |
| scara5 | 132.8 | 5.5 | -4.60 | 5.16E-05 | 0.0010 | 564500 | scavenger receptor class A, member 5 (putative) |
| desma | 16335.5 | 398.4 | -5.36 | 5.53E-05 | 0.0011 | 30148 | desmin a |
| tspan5a | 23.5 | 690.4 | 4.87 | 5.55E-05 | 0.0011 | 450026 | tetraspanin 5a |
| s100w | 1577.8 | 110.5 | -3.84 | 5.57E-05 | 0.0011 | 569958 | S100 calcium binding protein W |
| gypc | 190.2 | 10.7 | -4.15 | 5.60E-05 | 0.0011 | 335987 | glycophorin C (Gerbich blood group) |
| ap1m3 | 8.1 | 161.9 | 4.32 | 5.61E-05 | 0.0011 | 445244 | adaptor related protein complex 1 subunit mu 3 |
| pipox | 0.3 | 56.0 | 7.36 | 6.03E-05 | 0.0012 | 558601 | pipecolic acid oxidase |
| cpe | 50.6 | 968.5 | 4.26 | 6.03E-05 | 0.0012 | 407979 | carboxypeptidase E |
| dhh | 2.6 | 100.7 | 5.26 | 6.07E-05 | 0.0012 | 558147 | desert hedgehog signaling molecule |
| sh3gl2a | 7.3 | 279.1 | 5.25 | 6.27E-05 | 0.0012 | 394091 | SH3 domain containing GRB2 like 2a, endophilin A1 |
| zgc:112437 | 3.3 | 393.9 | 6.89 | 6.46E-05 | 0.0012 | 550468 | zgc:112437 |
| zgc:109782 | 21.1 | 366.4 | 4.12 | 6.49E-05 | 0.0012 | 777724 | zgc:109782 |
| rhbdl3 | 0.3 | 59.5 | 7.45 | 6.50E-05 | 0.0012 | 550128 | rhomboid, veinlet-like 3 (Drosophila) |
| mef2aa | 658.6 | 38.2 | -4.11 | 6.92E-05 | 0.0013 | 30564 | myocyte enhancer factor 2aa |
| camk2n2 | 12.5 | 191.5 | 3.94 | 7.02E-05 | 0.0013 | 436915 | calcium/calmodulin-dependent protein kinase II inhibitor 2 |
| crtc1b | 17.5 | 240.4 | 3.78 | 7.03E-05 | 0.0013 | 101886240 | CREB regulated transcription coactivator 1b |
| celf4 | 2.7 | 181.9 | 6.09 | 7.23E-05 | 0.0014 | 436835 | CUGBP, Elav-like family member 4 |
| wfdc1 | 140.9 | 2.1 | -6.05 | 7.28E-05 | 0.0014 | 560860 | WAP four-disulfide core domain 1 |
| itsn2a | 7.0 | 146.3 | 4.38 | 7.36E-05 | 0.0014 | 566412 | intersectin 2a |
| syngap1b | 13.6 | 253.2 | 4.22 | 7.48E-05 | 0.0014 | 100151149 | synaptic Ras GTPase activating protein 1b |
| slc25a51a | 6.2 | 132.0 | 4.41 | 7.94E-05 | 0.0015 | 393728 | solute carrier family 25 member 51a |
| drd4b | 2.0 | 81.2 | 5.37 | 8.34E-05 | 0.0015 | 503565 | dopamine receptor D4b |
| abat | 402.6 | 4788.7 | 3.57 | 8.40E-05 | 0.0015 | 378968 | 4-aminobutyrate aminotransferase |
| pisd | 24.7 | 253.0 | 3.35 | 9.06E-05 | 0.0016 | 553433 | phosphatidylserine decarboxylase |
| tac1 | 1.7 | 464.4 | 8.09 | 9.18E-05 | 0.0017 | 555634 | tachykinin precursor 1 |
| fam102ab | 7.9 | 153.8 | 4.28 | 9.40E-05 | 0.0017 | 767674 | family with sequence similarity 102 member Ab |
| dtnbb | 3.4 | 104.4 | 4.94 | 9.60E-05 | 0.0017 | 559786 | dystrobrevin, beta b |
| cd36 | 2.7 | 114.9 | 5.41 | 9.69E-05 | 0.0017 | 436636 | CD36 molecule (thrombospondin receptor) |
| slc1a7a | 0.0 | 42.0 | Inf | 9.91E-05 | 0.0018 | 100170783 | solute carrier family 1 member 7a |
| ttyh3b | 54.3 | 1214.3 | 4.48 | 0.00010 | 0.0018 | 798421 | tweety family member 3b |
| cyp2ad3 | 1532.5 | 94.9 | -4.01 | 0.00010 | 0.0019 | 569245 | cytochrome P450, family 2, subfamily AD, polypeptide 3 |
| stc1 | 2.7 | 523.6 | 7.61 | 0.00011 | 0.0019 | 751747 | stanniocalcin 1 |
| hoxc6a | 100.0 | 3.4 | -4.90 | 0.00011 | 0.0019 | 30346 | homeobox C6a |
| nlgn1 | 19.9 | 226.0 | 3.50 | 0.00012 | 0.0021 | 100002610 | neuroligin 1 |
| nrsn1l | 1.4 | 71.8 | 5.72 | 0.00012 | 0.0021 | 100007978 | neurensin 1-like |
| slc44a5b | 89.1 | 1107.3 | 3.64 | 0.00012 | 0.0022 | 558142 | solute carrier family 44 member 5b |
| chrnd | 235.7 | 3.4 | -6.10 | 0.00013 | 0.0022 | 404273 | cholinergic receptor, nicotinic, delta (muscle) |
| zgc:110716 | 38.5 | 0.0 | -Inf | 0.00013 | 0.0022 | 550248 | zgc:110716 |
| lrp11 | 14.1 | 255.7 | 4.18 | 0.00013 | 0.0022 | 569216 | low density lipoprotein receptor-related protein 11 |
| l1camb | 4.8 | 107.7 | 4.48 | 0.00013 | 0.0022 | 30656 | L1 cell adhesion molecule, paralog b |
| carmil2 | 11.0 | 167.4 | 3.93 | 0.00014 | 0.0024 | 567758 | capping protein regulator and myosin 1 linker 2 |
| parvab | 125.3 | 5.8 | -4.45 | 0.00014 | 0.0024 | 567123 | parvin, alpha b |
| pax3a | 2.2 | 177.0 | 6.32 | 0.00014 | 0.0024 | 30532 | paired box 3a |
| myl9a | 1324.0 | 111.6 | -3.57 | 0.00014 | 0.0024 | 450006 | myosin, light chain 9a, regulatory |
| hoxa9b | 932.0 | 73.0 | -3.67 | 0.00014 | 0.0025 | 58048 | homeobox A9b |
| cfl2 | 2704.9 | 118.9 | -4.51 | 0.00015 | 0.0026 | 403001 | cofilin 2 (muscle) |
| nrn1b | 1.8 | 96.2 | 5.71 | 0.00015 | 0.0026 | 449839 | neuritin 1b |
| vox | 0.0 | 37.1 | Inf | 0.00017 | 0.0028 | 64807 | ventral homeobox |
| ptprfa | 48.2 | 722.8 | 3.91 | 0.00017 | 0.0029 | 140819 | protein tyrosine phosphatase receptor type Fa |
| six1b | 1047.3 | 66.7 | -3.97 | 0.00017 | 0.0029 | 404627 | SIX homeobox 1b |
| crtap | 1162.6 | 86.0 | -3.76 | 0.00017 | 0.0029 | 406626 | cartilage associated protein |
| cyp2p7 | 145.0 | 1.9 | -6.29 | 0.00017 | 0.0029 | 100034406 | cytochrome P450, family 2, subfamily P, polypeptide 7 |
| hspb11 | 379.5 | 13.2 | -4.84 | 0.00018 | 0.0030 | 796767 | heat shock protein, alpha-crystallin-related, b11 |
| lrrc4ca | 2.6 | 390.0 | 7.22 | 0.00018 | 0.0030 | 553785 | leucine rich repeat containing 4C, genome duplicate a |
| guk1b | 0.0 | 46.6 | Inf | 0.00018 | 0.0030 | 393697 | guanylate kinase 1b |
| mmp2 | 7598.7 | 452.4 | -4.07 | 0.00018 | 0.0031 | 337179 | matrix metallopeptidase 2 |
| ank1a | 216.6 | 12.5 | -4.11 | 0.00019 | 0.0032 | 449796 | ankyrin 1, erythrocytic a |
| en1b | 720.0 | 41.2 | -4.13 | 0.00019 | 0.0032 | 541371 | engrailed homeobox 1b |
| fitm1 | 115.4 | 5.3 | -4.45 | 0.00022 | 0.0035 | 503747 | fat storage-inducing transmembrane protein 1 |
| elavl4 | 42.9 | 1032.5 | 4.59 | 0.00022 | 0.0036 | 30737 | ELAV like neuron-specific RNA binding protein 4 |
| fstl4 | 20.1 | 214.0 | 3.41 | 0.00022 | 0.0036 | 571664 | follistatin-like 4 |
| sema3ga | 24.3 | 343.0 | 3.82 | 0.00023 | 0.0038 | 544659 | sema domain, immunoglobulin domain (Ig), short basic domain, secreted, (semaphorin) 3Ga |
| jpt1a | 19.1 | 222.5 | 3.55 | 0.00024 | 0.0038 | 100037360 | Jupiter microtubule associated homolog 1a |
| cyp2n13 | 467.2 | 14.1 | -5.05 | 0.00025 | 0.0041 | 492484 | cytochrome P450, family 2, subfamily N, polypeptide 13 |
| si:dkey-9i23.16 | 285.9 | 17.9 | -4.00 | 0.00026 | 0.0042 | 571915 | si:dkey-9i23.16 |
| gpm6bb | 527.3 | 6950.6 | 3.72 | 0.00026 | 0.0042 | 503756 | glycoprotein M6Bb |
| hspb15 | 5.2 | 102.9 | 4.31 | 0.00027 | 0.0043 | 555589 | heat shock protein, alpha-crystallin-related, b15 |
| si:dkey-91i10.2 | 1.3 | 57.7 | 5.49 | 0.00027 | 0.0043 | 555224 | si:dkey-91i10.2 |
| insm1b | 73.6 | 1164.1 | 3.98 | 0.00027 | 0.0043 | 323882 | insulinoma-associated 1b |
| egr4 | 64.4 | 5058.1 | 6.30 | 0.00029 | 0.0045 | 795099 | early growth response 4 |
| rps8b | 7.8 | 125.9 | 4.02 | 0.00029 | 0.0046 | 100000836 | ribosomal protein S8b |
| mfge8b | 554.5 | 26.1 | -4.41 | 0.00030 | 0.0046 | 558580 | milk fat globule-EGF factor 8 protein b |
| zgc:152904 | 11.1 | 148.2 | 3.74 | 0.00030 | 0.0047 | 767777 | zgc:152904 |
| ntm | 4.4 | 94.8 | 4.42 | 0.00031 | 0.0047 | 678534 | neurotrimin |
| snap25b | 0.8 | 191.3 | 7.82 | 0.00031 | 0.0048 | 30711 | synaptosome associated protein 25b |
| egr2a | 2056.5 | 157.8 | -3.70 | 0.00031 | 0.0048 | 368241 | early growth response 2a |
| lgi1b | 4.3 | 146.9 | 5.10 | 0.00034 | 0.0052 | 654829 | leucine-rich, glioma inactivated 1b |
| timd4 | 14.5 | 387.5 | 4.74 | 0.00034 | 0.0053 | 100142639 | T cell immunoglobulin and mucin domain containing 4 |
| prom1a | 7.5 | 1079.9 | 7.18 | 0.00035 | 0.0054 | 322857 | prominin 1a |
| efemp2a | 2439.8 | 109.9 | -4.47 | 0.00035 | 0.0054 | 494044 | EGF containing fibulin extracellular matrix protein 2a |
| eva1bb | 1511.3 | 143.9 | -3.39 | 0.00035 | 0.0054 | 798034 | eva-1 homolog Bb (C. elegans) |
| zgc:162879 | 2.1 | 69.3 | 5.02 | 0.00036 | 0.0055 | 572256 | zgc:162879 |
| tdgf1 | 50.8 | 1.0 | -5.66 | 0.00036 | 0.0055 | 30304 | teratocarcinoma-derived growth factor 1 |
| rab42a | 0.6 | 48.4 | 6.43 | 0.00036 | 0.0055 | 406315 | RAB42, member RAS oncogene family a |
| slc4a3 | 14.5 | 172.3 | 3.57 | 0.00036 | 0.0055 | 100333073 | solute carrier family 4 member 3 |
| chrd | 240.2 | 9.5 | -4.66 | 0.00036 | 0.0055 | 30161 | chordin |
| baalca | 0.0 | 32.5 | Inf | 0.00038 | 0.0057 | 572429 | BAALC binder of MAP3K1 and KLF4 a |
| cacnb4a | 1.3 | 53.4 | 5.38 | 0.00038 | 0.0057 | 562422 | calcium channel, voltage-dependent, beta 4a subunit |
| wif1 | 1007.2 | 85.6 | -3.56 | 0.00039 | 0.0058 | 30476 | wnt inhibitory factor 1 |
| nptnb | 3.2 | 668.1 | 7.69 | 0.00039 | 0.0058 | 403006 | neuroplastin b |
| zfp36l2 | 1409.1 | 137.9 | -3.35 | 0.00040 | 0.0060 | 333941 | zinc finger protein 36, C3H type-like 2 |
| sox8a | 1.1 | 52.7 | 5.57 | 0.00041 | 0.0060 | 102216265 | SRY-box transcription factor 8a |
| cntfr | 7.8 | 125.3 | 4.00 | 0.00041 | 0.0061 | 368438 | ciliary neurotrophic factor receptor |
| atp2b3a | 11.8 | 911.8 | 6.28 | 0.00042 | 0.0062 | 436745 | ATPase plasma membrane Ca2+ transporting 3a |
| cd8a | 206.0 | 2706.5 | 3.72 | 0.00042 | 0.0062 | 677754 | CD8a molecule |
| adprhl1 | 1222.3 | 32.4 | -5.24 | 0.00042 | 0.0062 | 450012 | ADP-ribosylhydrolase like 1 |
| fgf13b | 6.3 | 678.3 | 6.75 | 0.00043 | 0.0063 | 403003 | fibroblast growth factor 13b |
| zgc:172282 | 1.3 | 52.5 | 5.36 | 0.00043 | 0.0063 | 100003690 | zgc:172282 |
| mamdc2a | 241.7 | 12.8 | -4.24 | 0.00043 | 0.0063 | 556154 | MAM domain containing 2a |
| efna3b | 11.6 | 171.1 | 3.88 | 0.00043 | 0.0063 | 117506 | ephrin-A3b |
| tp63 | 75.4 | 2.8 | -4.74 | 0.00044 | 0.0064 | 260407 | tumor protein p63 |
| cadm1a | 41.2 | 987.0 | 4.58 | 0.00044 | 0.0065 | 569931 | cell adhesion molecule 1a |
| grin1b | 2.6 | 206.0 | 6.33 | 0.00044 | 0.0065 | 100005675 | glutamate receptor, ionotropic, N-methyl D-aspartate 1b |
| zgc:110339 | 5630.8 | 379.1 | -3.89 | 0.00046 | 0.0067 | 550490 | zgc:110339 |
| tcf15 | 1731.5 | 135.4 | -3.68 | 0.00046 | 0.0067 | 30159 | transcription factor 15 |
| ccdc149a | 6.1 | 98.7 | 4.00 | 0.00047 | 0.0068 | 449999 | coiled-coil domain containing 149a |
| adcy5 | 16.2 | 173.3 | 3.42 | 0.00048 | 0.0069 | 562619 | adenylate cyclase 5 |
| trpc4a | 413.1 | 3.3 | -6.97 | 0.00051 | 0.0073 | 102725537 | transient receptor potential cation channel, subfamily C, member 4a |
| nat8l | 2.6 | 119.9 | 5.55 | 0.00052 | 0.0074 | 564754 | N-acetyltransferase 8-like |
| si:dkey-20d21.12 | 100.4 | 5.7 | -4.15 | 0.00052 | 0.0074 | 556245 | si:dkey-20d21.12 |
| smtnl | 100.7 | 5.8 | -4.12 | 0.00052 | 0.0074 | 399690 | smoothelin, like |
| slc35g2b | 1.5 | 56.9 | 5.21 | 0.00052 | 0.0074 | 541438 | solute carrier family 35 member G2b |
| fam43b | 8.1 | 306.1 | 5.23 | 0.00052 | 0.0074 | 447808 | family with sequence similarity 43 member B |
| ppm1lb | 16.5 | 178.2 | 3.43 | 0.00053 | 0.0075 | 767640 | protein phosphatase, Mg2+/Mn2+ dependent, 1Lb |
| sncb | 0.7 | 98.4 | 7.17 | 0.00055 | 0.0078 | 393944 | synuclein, beta |
| kcnk15 | 0.0 | 29.5 | Inf | 0.00057 | 0.0079 | 100000132 | potassium channel, subfamily K, member 15 |
| zgc:92912 | 0.0 | 29.1 | Inf | 0.00058 | 0.0081 | 436805 | zgc:92912 |
| hoxa2b | 142.8 | 11.3 | -3.66 | 0.00061 | 0.0084 | 30325 | homeobox A2b |
| coro6 | 97.8 | 0.0 | -Inf | 0.00066 | 0.0090 | 393367 | coronin 6 |
| trim35-24 | 126.4 | 10.9 | -3.54 | 0.00066 | 0.0091 | 557392 | tripartite motif containing 35-24 |
| hnmt | 5.0 | 88.7 | 4.16 | 0.00067 | 0.0091 | 445242 | histamine N-methyltransferase |
| cadm1b | 0.0 | 27.8 | Inf | 0.00067 | 0.0092 | 562183 | cell adhesion molecule 1b |
| gpr186 | 0.3 | 1364.0 | 11.97 | 0.00068 | 0.0093 | 796164 | G protein-coupled receptor 186 |
| elovl6 | 5.4 | 103.0 | 4.25 | 0.00069 | 0.0093 | 317738 | ELOVL fatty acid elongase 6 |
| pgam2 | 176.7 | 9.1 | -4.27 | 0.00069 | 0.0094 | 572733 | phosphoglycerate mutase 2 (muscle) |
| sox1b | 1.7 | 61.2 | 5.18 | 0.00072 | 0.0096 | 562710 | SRY-box transcription factor 1b |
| glra2 | 0.0 | 32.6 | Inf | 0.00072 | 0.0097 | 793646 | glycine receptor, alpha 2 |
| kcnn1a | 6.7 | 104.2 | 3.96 | 0.00072 | 0.0097 | 563352 | potassium intermediate/small conductance calcium-activated channel, subfamily N, member 1a |
| zgc:136605 | 117.8 | 9.7 | -3.60 | 0.00073 | 0.0097 | 664695 | zgc:136605 |
