# Supplementary material for: Defining function of wild-type and three patient-specific TP53 mutations in a zebrafish model of embryonal rhabdomyosarcoma
Source: eLife. 2023 Jun 2;12:e68221. doi: 10.7554/eLife.68221 (PMC10322150; doi:10.7554/eLife.68221)
Supplement: Supplementary file 3. [file elife-68221-supp3.docx]

**Supplementary Table 3.**

| **Symbol** | **baseMean Trunk** | **baseMean Head** | **log2FC** | **pval** | **padj** | **ENTREZID** | **GENENAME** |
| --- | --- | --- | --- | --- | --- | --- | --- |
| LOC100334800 | 44.8 | 5904.9 | 7.042 | 2.49E-20 | 1.79E-16 | 100334800 | microfibril-associated glycoprotein 4-like |
| LOC566119 | 10.4 | 1881.5 | 7.505 | 2.87E-20 | 1.79E-16 | 566119 | microfibril-associated glycoprotein 4-like |
| jac7 | 5.8 | 998.7 | 7.418 | 2.90E-16 | 1.21E-12 | 797049 | jacalin 7 |
| dlx4b | 0.0 | 334.2 | Inf | 5.62E-13 | 1.76E-09 | 30581 | distal-less homeobox 4b |
| ptch2 | 28.2 | 913.2 | 5.015 | 1.55E-10 | 3.88E-07 | 30181 | patched 2 |
| dap | 0.0 | 170.1 | Inf | 3.73E-09 | 7.77E-06 | 58093 | death-associated protein |
| tbx1 | 6.3 | 389.3 | 5.947 | 8.81E-09 | 1.57E-05 | 368206 | T-box 1 |
| eomesa | 138.5 | 2059.4 | 3.894 | 2.94E-08 | 4.60E-05 | 64603 | eomesodermin homolog a |
| mef2b | 102.6 | 1552.2 | 3.920 | 4.24E-08 | 5.89E-05 | 558073 | myocyte enhancer factor 2b |
| dkk3b | 2633.0 | 95.4 | -4.787 | 5.12E-08 | 6.40E-05 | 100038765 | dickkopf WNT signaling pathway inhibitor 3b |
| apoa2 | 141.5 | 1869.7 | 3.724 | 1.27E-07 | 0.00014 | 322327 | apolipoprotein A-II |
| si:dkey-9i23.4 | 16.1 | 466.9 | 4.860 | 1.47E-07 | 0.00015 | 571855 | si:dkey-9i23.4 |
| apoa1b | 661.6 | 7701.9 | 3.541 | 1.52E-07 | 0.00015 | 100101640 | apolipoprotein A-Ib |
| chia.5 | 14.8 | 430.6 | 4.866 | 1.95E-07 | 0.00017 | 100003900 | chitinase, acidic.5 |
| si:ch1073-376c22.1 | 84.7 | 1274.0 | 3.911 | 2.35E-07 | 0.00020 | 100000126 | si:ch1073-376c22.1 |
| si:ch1073-67j19.1 | 85.9 | 1215.0 | 3.822 | 3.10E-07 | 0.00024 | 100332348 | si:ch1073-67j19.1 |
| npsn | 6606.1 | 65955.1 | 3.320 | 4.36E-07 | 0.00031 | 404039 | nephrosin |
| foxf2a | 0.0 | 97.3 | Inf | 4.42E-07 | 0.00031 | 407681 | forkhead box F2a |
| ovol1a | 1.6 | 150.4 | 6.525 | 6.44E-07 | 0.00042 | 792456 | ovo-like zinc finger 1a |
| lect2l | 4684.7 | 46531.8 | 3.312 | 8.91E-07 | 0.00056 | 567278 | leukocyte cell-derived chemotaxin 2 like |
| chia.6 | 120.3 | 1436.2 | 3.577 | 1.10E-06 | 0.00066 | 322420 | chitinase, acidic.6 |
| mpx | 1792.6 | 18086.4 | 3.335 | 1.24E-06 | 0.00069 | 337514 | myeloid-specific peroxidase |
| b3gnt5b | 74.4 | 932.9 | 3.649 | 1.27E-06 | 0.00069 | 791470 | UDP-GlcNAc:betaGal beta-1,3-N-acetylglucosaminyl-transferase 5b |
| grin2ca | 609.3 | 18.7 | -5.028 | 1.45E-06 | 0.00075 | 100003342 | glutamate receptor, ionotropic, N-methyl D-aspartate 2Ca |
| fstl3 | 2339.6 | 105.2 | -4.475 | 1.59E-06 | 0.00079 | 557352 | follistatin-like 3 (secreted glycoprotein) |
| dlx3b | 86.6 | 852.3 | 3.299 | 1.81E-06 | 0.00087 | 30585 | distal-less homeobox 3b |
| prf1.2 | 38.9 | 581.0 | 3.902 | 2.05E-06 | 0.00095 | 103909237 | perforin 1.2 |
| tm4sf4 | 9.2 | 287.0 | 4.962 | 2.15E-06 | 0.00096 | 445095 | transmembrane 4 L six family member 4 |
| il1b | 895.1 | 8862.8 | 3.308 | 2.74E-06 | 0.0012 | 405770 | interleukin 1, beta |
| lyz | 1597.0 | 15221.9 | 3.253 | 3.07E-06 | 0.0013 | 677744 | lysozyme |
| selp | 116.7 | 1301.5 | 3.479 | 3.12E-06 | 0.0013 | 796481 | selectin P |
| cpa5 | 1584.5 | 14949.6 | 3.238 | 3.22E-06 | 0.0013 | 246092 | carboxypeptidase A5 |
| prkcda | 314.6 | 2855.6 | 3.182 | 3.47E-06 | 0.0013 | 334571 | protein kinase C, delta a |
| zgc:77112 | 231.7 | 2180.3 | 3.234 | 5.84E-06 | 0.0021 | 406459 | zgc:77112 |
| s1pr4 | 223.3 | 2035.8 | 3.189 | 5.91E-06 | 0.0021 | 402857 | sphingosine-1-phosphate receptor 4 |
| rab3db | 30.5 | 474.8 | 3.960 | 7.86E-06 | 0.0027 | 436952 | RAB3D, member RAS oncogene family, b |
| fabp10a | 59.7 | 670.4 | 3.490 | 7.99E-06 | 0.0027 | 171481 | fatty acid binding protein 10a, liver basic |
| LOC100003647 | 115.9 | 1183.5 | 3.353 | 8.16E-06 | 0.0027 | 100003647 | three-finger protein 5 |
| timp4.2 | 142.8 | 1405.7 | 3.299 | 8.51E-06 | 0.0027 | 108004535 | TIMP metallopeptidase inhibitor 4, tandem duplicate 2 |
| csf3r | 277.5 | 2381.8 | 3.102 | 9.44E-06 | 0.0029 | 100134935 | colony stimulating factor 3 receptor (granulocyte) |
| itln1 | 100.6 | 1050.8 | 3.385 | 1.05E-05 | 0.0032 | 767684 | intelectin 1 |
| myh11a | 609.2 | 4941.6 | 3.020 | 1.31E-05 | 0.0039 | 554168 | myosin, heavy chain 11a, smooth muscle |
| atp1a1a.4 | 20.1 | 326.4 | 4.020 | 1.34E-05 | 0.0039 | 64615 | ATPase Na+/K+ transporting subunit alpha 1a, tandem duplicate 4 |
| myf6 | 889.3 | 48.2 | -4.207 | 1.41E-05 | 0.0039 | 404208 | myogenic factor 6 |
| sult5a1 | 75.5 | 751.0 | 3.315 | 1.41E-05 | 0.0039 | 767718 | sulfotransferase family 5A, member 1 |
| hyal3 | 101.1 | 946.6 | 3.227 | 1.50E-05 | 0.0041 | 100004557 | hyaluronidase 3 |
| tuba7l | 589.8 | 3619.4 | 2.617 | 1.62E-05 | 0.0043 | 431777 | tubulin, alpha 7 like |
| atp1a3a | 408.0 | 3221.3 | 2.981 | 1.72E-05 | 0.0045 | 64610 | ATPase Na+/K+ transporting subunit alpha 3a |
| stc1l | 2.9 | 136.6 | 5.536 | 1.75E-05 | 0.0045 | 393511 | stanniocalcin 1, like |
| ociad2 | 3297.6 | 306.7 | -3.427 | 1.83E-05 | 0.0046 | 558261 | OCIA domain containing 2 |
| dnah9 | 173.6 | 1179.6 | 2.764 | 2.13E-05 | 0.0052 | 266676 | dynein, axonemal, heavy chain 9 |
| enkur | 120.7 | 873.9 | 2.856 | 2.60E-05 | 0.0063 | 402794 | enkurin, TRPC channel interacting protein |
| diras1a | 341.6 | 11.8 | -4.856 | 3.06E-05 | 0.0071 | 327577 | DIRAS family, GTP-binding RAS-like 1a |
| piwil1 | 221.2 | 1406.7 | 2.669 | 3.08E-05 | 0.0071 | 368200 | piwi-like RNA-mediated gene silencing 1 |
| ch25hl2 | 128.1 | 1113.8 | 3.120 | 3.25E-05 | 0.0074 | 100136851 | cholesterol 25-hydroxylase like 2 |
| lingo4b | 231.4 | 5.9 | -5.294 | 3.43E-05 | 0.0076 | 559074 | leucine rich repeat and Ig domain containing 4b |
| neu4 | 22.3 | 347.0 | 3.963 | 3.76E-05 | 0.0082 | 553569 | sialidase 4 |
| erg | 64.7 | 635.0 | 3.296 | 3.81E-05 | 0.0082 | 494073 | ETS transcription factor ERG |
| fes | 95.0 | 832.6 | 3.131 | 3.89E-05 | 0.0082 | 566739 | FES proto-oncogene, tyrosine kinase |
| si:dkey-9i23.5 | 0.4 | 61.9 | 7.201 | 4.21E-05 | 0.0085 | 571862 | si:dkey-9i23.5 |
| si:ch211-66e2.3 | 108.7 | 873.9 | 3.007 | 4.23E-05 | 0.0085 | 100141487 | si:ch211-66e2.3 |
| alox5ap | 108.9 | 935.8 | 3.103 | 4.24E-05 | 0.0085 | 337376 | arachidonate 5-lipoxygenase-activating protein |
| si:ch211-251b21.1 | 1161.5 | 119.9 | -3.276 | 4.43E-05 | 0.0088 | 571720 | si:ch211-251b21.1 |
| illr4 | 143.2 | 1144.2 | 2.998 | 4.60E-05 | 0.0089 | 559737 | immune-related, lectin-like receptor 4 |
| thbs3b | 3822.4 | 292.9 | -3.706 | 4.62E-05 | 0.0089 | 571317 | thrombospondin 3b |
| nkl.1 | 5.0 | 153.3 | 4.925 | 5.05E-05 | 0.0095 | 103909014 | NK-lysin tandem duplicate 1 |
| zgc:92360 | 170.5 | 1289.7 | 2.919 | 5.25E-05 | 0.0095 | 436988 | zgc:92360 |
| hbaa1 | 1067.1 | 109.1 | -3.290 | 5.27E-05 | 0.0095 | 30507 | hemoglobin, alpha adult 1 |
| mrc1b | 1878.4 | 12939.4 | 2.784 | 5.27E-05 | 0.0095 | 559502 | mannose receptor, C type 1b |
