# Supplementary material for: Defining function of wild-type and three patient-specific TP53 mutations in a zebrafish model of embryonal rhabdomyosarcoma
Source: eLife. 2023 Jun 2;12:e68221. doi: 10.7554/eLife.68221 (PMC10322150; doi:10.7554/eLife.68221)
Supplement: Supplementary file 4. [file elife-68221-supp4.docx]

| **Name** | **Sequence** |
| --- | --- |
| 18s-QRT-1-F | TCGCTAGTTGGCATCGTTTATG |
| 18s-QRT-1-R | CGGAGGTTCGAAGACGATCA |
| *hKRAS*-QRT-F-137 | TTGATGGAGAAACCTGTCTCTTGG |
| *hKRAS*-QRT-R-247 | CAAATACACAAAGAAAGCCCTCCC |
| *dusp4*-14F | ACGAACTTTGCGAAATGGAC |
| dusp4-95R | CTGCAATCCAGAAGCAGACA |
| *TP53* fwd | CACCGTGTGCTGGAATTCGGCTTA |
| *TP53P153* XO Rev | GTAGATGGCCATGGCGCGGACGCGGGTGCCCGGGGGTGTGGAATCAACCCACAGCTGCAC |
| *TP53P153* XO Fwd | GTGCAGCTGTGGGTTGATTCCACACCCCCGGGCACCCGCGTCCGCGCCATGGCCATCTAC |
| *TP53* rev | ATCTGCAGAATTCGGCTTTC |
| *TP53C176F* XO Rev | TCGCTATCTGAGCAGCGCTCATGGTGGGGGAAGCGCCTCACAACCTCCGTCATGTGCTGTGAC |
| *TP53C176F* XO Fwd | GTCACAGCACATGACGGAGGTTGTGAGGCGCTTCCCCCACCATGAGCGCTGCTCAGATAGCGA |
| *TP53Y220C* XO Fwd | CACTTTTCGACATAGTGTGGTGGTGCCCTGTGAGCCGCCTGAGGTTGGCTCTGACTGTAC |
| *TP53Y220C* XO Rev | GTACAGTCAGAGCCAACCTCAGGCGGCTCACAGGGCACCACCACACTATGTCGAAAAGTG |
| \| *baxa*-F1 \| CGTCGGGTGGAGGCGATACG \| \| --- \| --- \| | CGTCGGGTGGAGGCGATACG |
| *baxa*-R1 | GAGTCGGCTGAAGATTAGAGTT |
| *bbc3-*F1 | CATCTTCACATGCACACGGC |
| *bbc3-*R1 | CCCAGAATCGTGATGTCCTGA |
| *gadd45ab*-F1 | AACCGTGTGGAGATAACGCA |
| *gadd45ab*-R1 | TAAGCGACTTCGCTGCTTCA |
| *noxa*-L-F1 | ATGGCGAAGAAAGAGCAAAC |
| *noxa*-L-R1 | CTCATCGCTTCCCCTCCA |
| *cdkn1a*-F1 | CCAGCTTCAGGTGTTCCTCAG |
| *cdkn1a*-R1 | GTGAACGTAGGATCCGCTTGT |
| *tp53-*F1 | ATCCGGGCAATCCGAAAGTC |
| *tp53*-R1 | GTGAACGTAGGATCCGCTTGT |
| *kdrEx12*-13-gen-F1 | GCACCAAGTTTCTGTATTGAGG |
| kdrEx12-13-gen-R1 | ACTAAGGACCTACCTCTGAGTC |
|  |  |
|  |  |
