# Supplementary material for: Defining function of wild-type and three patient-specific TP53 mutations in a zebrafish model of embryonal rhabdomyosarcoma
Source: eLife. 2023 Jun 2;12:e68221. doi: 10.7554/eLife.68221 (PMC10322150; doi:10.7554/eLife.68221)
Supplement: Supplementary file 5. [file elife-68221-supp5.docx]

| **Antibody** | **Catalog No.** | **Vendor** |
| --- | --- | --- |
| anti-DsRed antibody | 632496 | Takara Bio |
| anti-p53 antibody (human) | ab1101 | Cell Signaling Technology |
| anti-p53 antibody (fish) | ab77813 | Abcam |
| anti-GAPDH antibody | 2118 | Cell Signaling Technology |
| anti-β-actin antibody (human) | A00702-40 | GenScript |
| anti-p21 antibody (human) | 2946 | Cell Signaling Technology |
| anti-β-tubulin antibody (human) | ab6046 | Abcam |
| anti-Sox10 (zebrafish) | GTX128374 | GeneTex |
| anti-Gfap (zebrafish) | G3893 | Sigma Aldrich |
| anti-myosin heavy chain (MF20) | MF 20 | DSHB |
| anti-VEGF Receptor 2 (KDR) (human) | 12556 | Cell Signaling Technology |
| anti-phosphohistoneH3 (Ser10) | 9701S | Cell Signaling Technology |
| HRP anti-rabbit | 7074 | Cell Signaling Technology |
| HRP anti-mouse | NA93IV | GE Healthcare |
