# Supplementary material for: Defining function of wild-type and three patient-specific TP53 mutations in a zebrafish model of embryonal rhabdomyosarcoma
Source: eLife. 2023 Jun 2;12:e68221. doi: 10.7554/eLife.68221 (PMC10322150; doi:10.7554/eLife.68221)
Supplement: Supplementary file 6. [file elife-68221-supp6.docx]

|  | **Catalog No.** | **Vendor** |
| --- | --- | --- |
| Click-iT™ EdU Alexa Fluor™ 647 Flow Cytometry Assay Kit | C10419 | ThermoFisher Scientific |
| Annexin V, Alexa Fluor™ 647 conjugate | A23204 | Invitrogen |
| IncuCyte® Caspase-3/7 Green Apoptosis Assay Reagent | 4440 | Sartorius |
| ZMC1 | HY-18634 | MedChemExpress |
