# Supplementary material for: Defining function of wild-type and three patient-specific TP53 mutations in a zebrafish model of embryonal rhabdomyosarcoma
Source: eLife. 2023 Jun 2;12:e68221. doi: 10.7554/eLife.68221 (PMC10322150; doi:10.7554/eLife.68221)
Supplement: Source data 1. [file elife-68221-data1.pptx]

## Slide 1
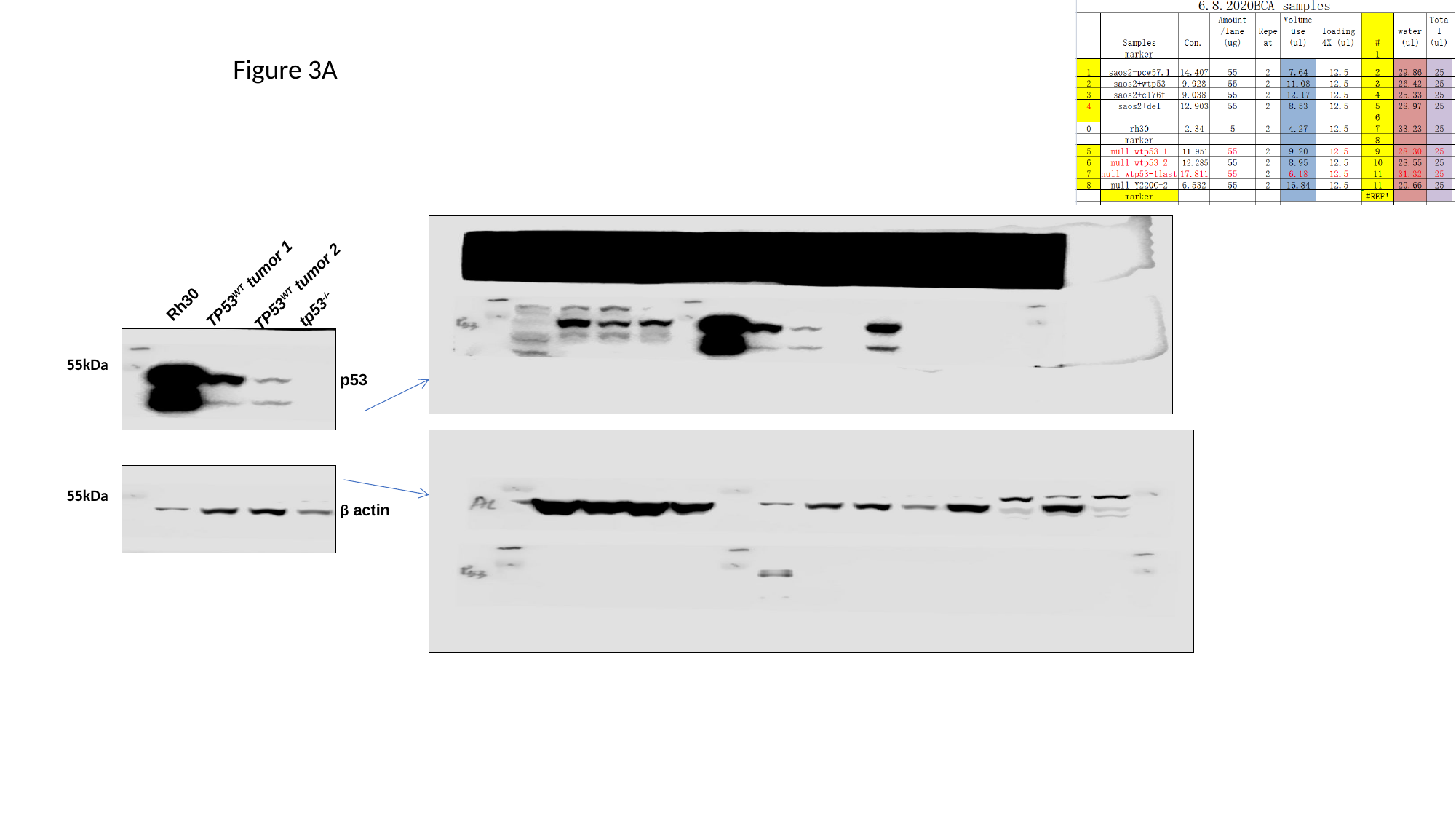

Figure 3A
TP53WT tumor 1
TP53WT tumor 2
Rh30
tp53-/-
55kDa
p53
55kDa
β actin

## Slide 2
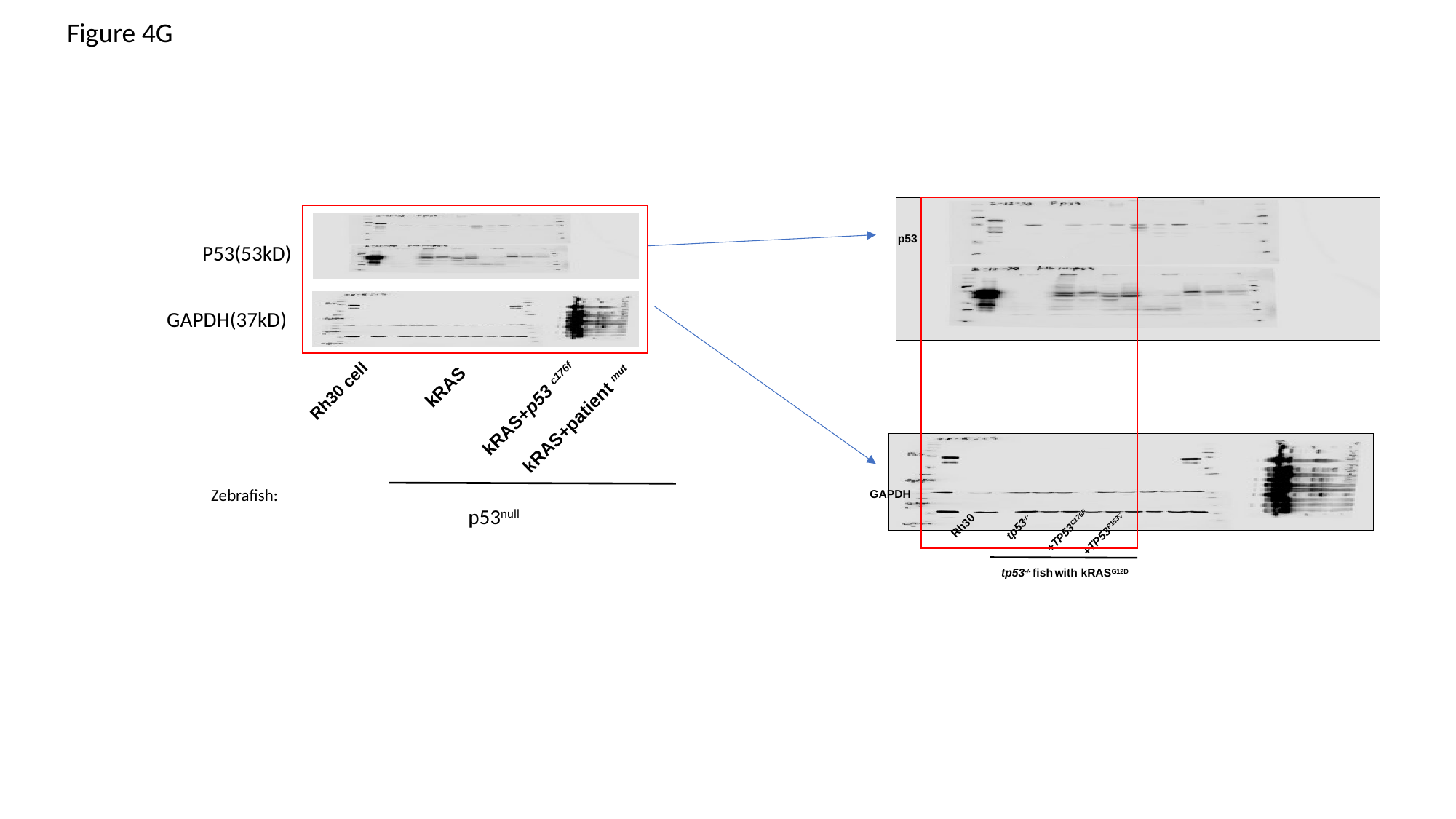

Figure 4G
p53
P53(53kD)
GAPDH(37kD)
kRAS
Rh30 cell
kRAS+p53 c176f
kRAS+patient mut
Zebrafish:
GAPDH
p53null
Rh30
+TP53C176F
+TP53P153▽
tp53-/-
tp53-/- fish with kRASG12D

## Slide 3
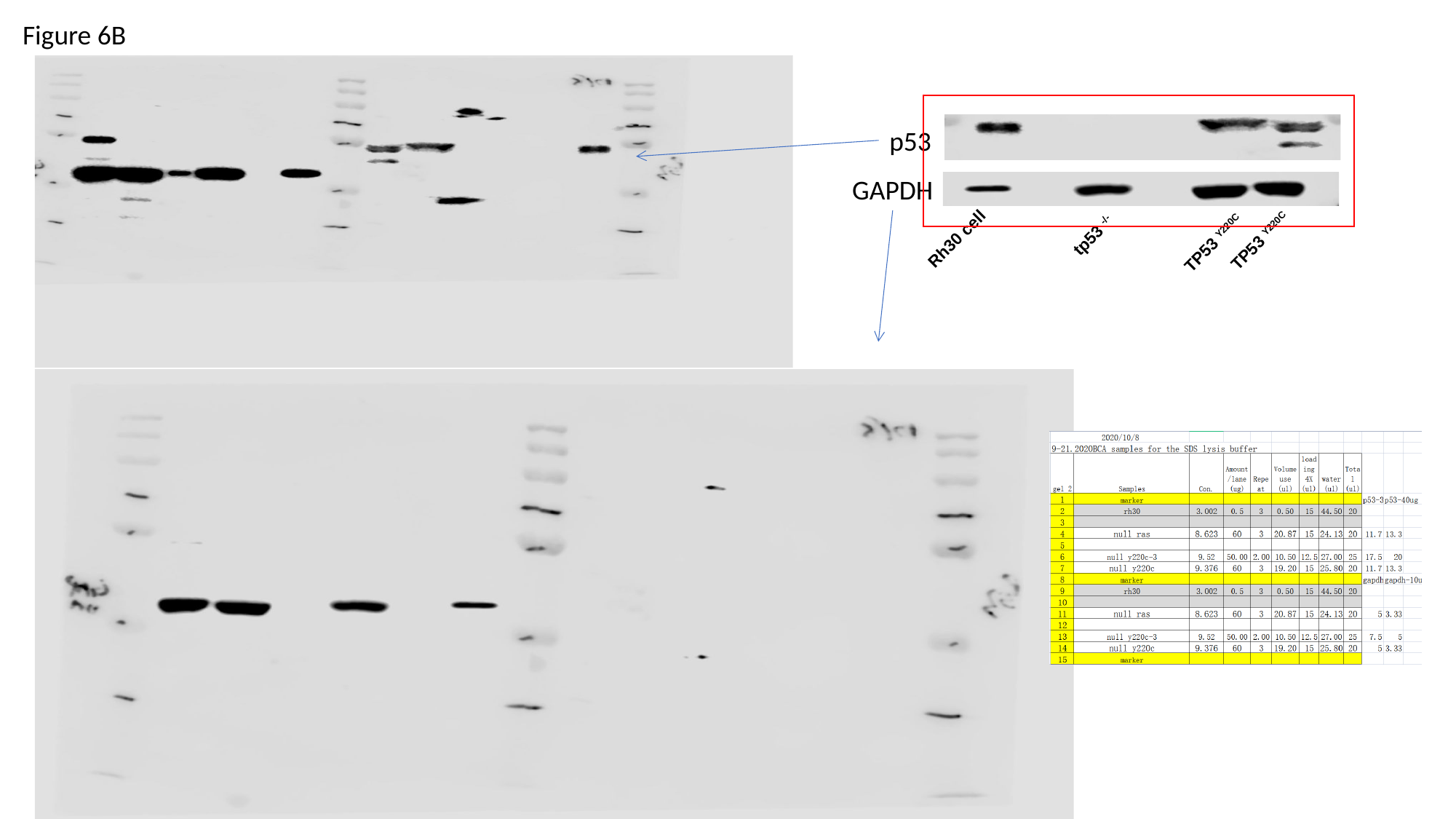

Figure 6B
p53
GAPDH
Rh30 cell
TP53 Y220C
TP53 Y220C
tp53 -/-

## Slide 4
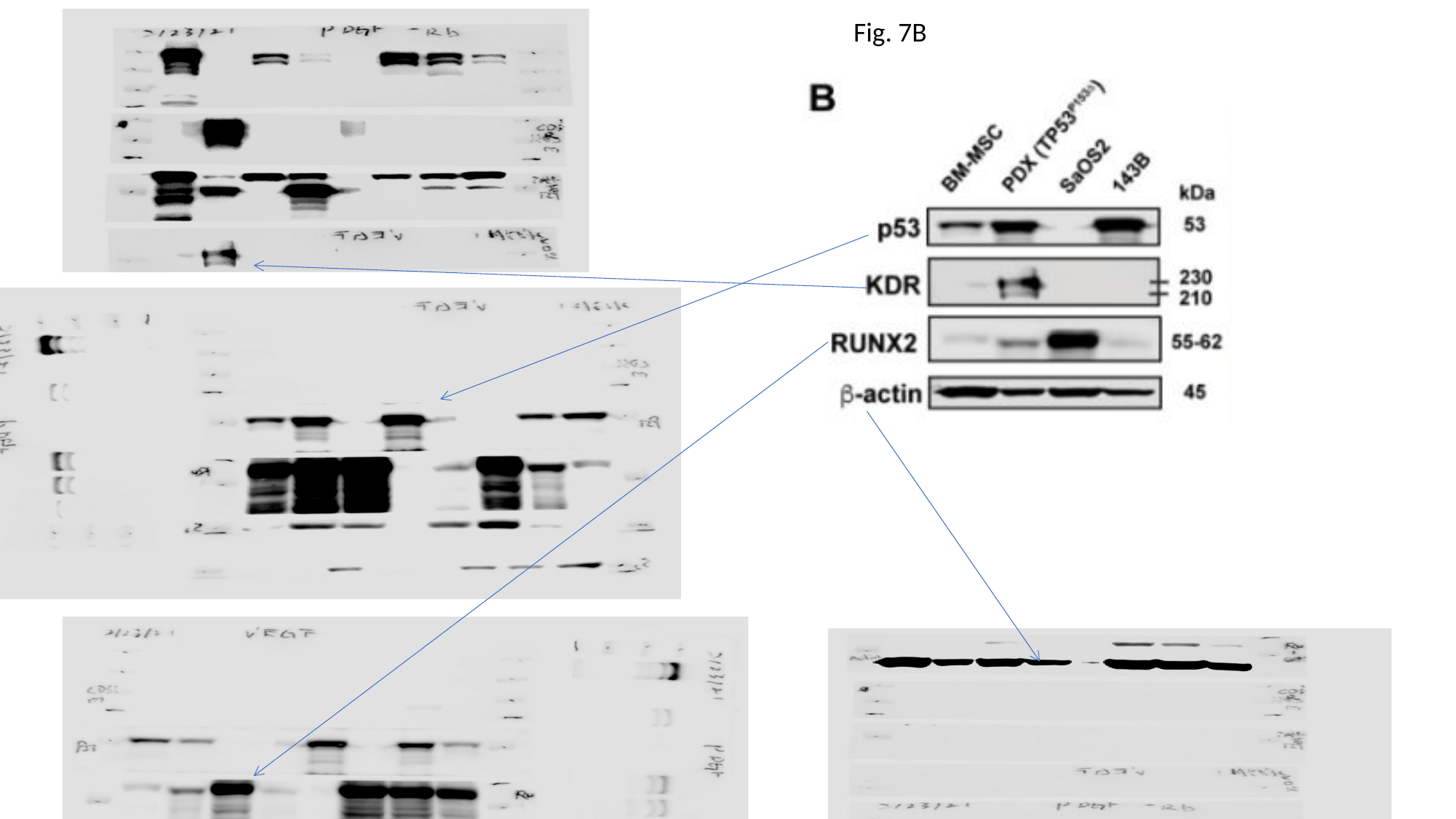

Fig. 7B

## Slide 5
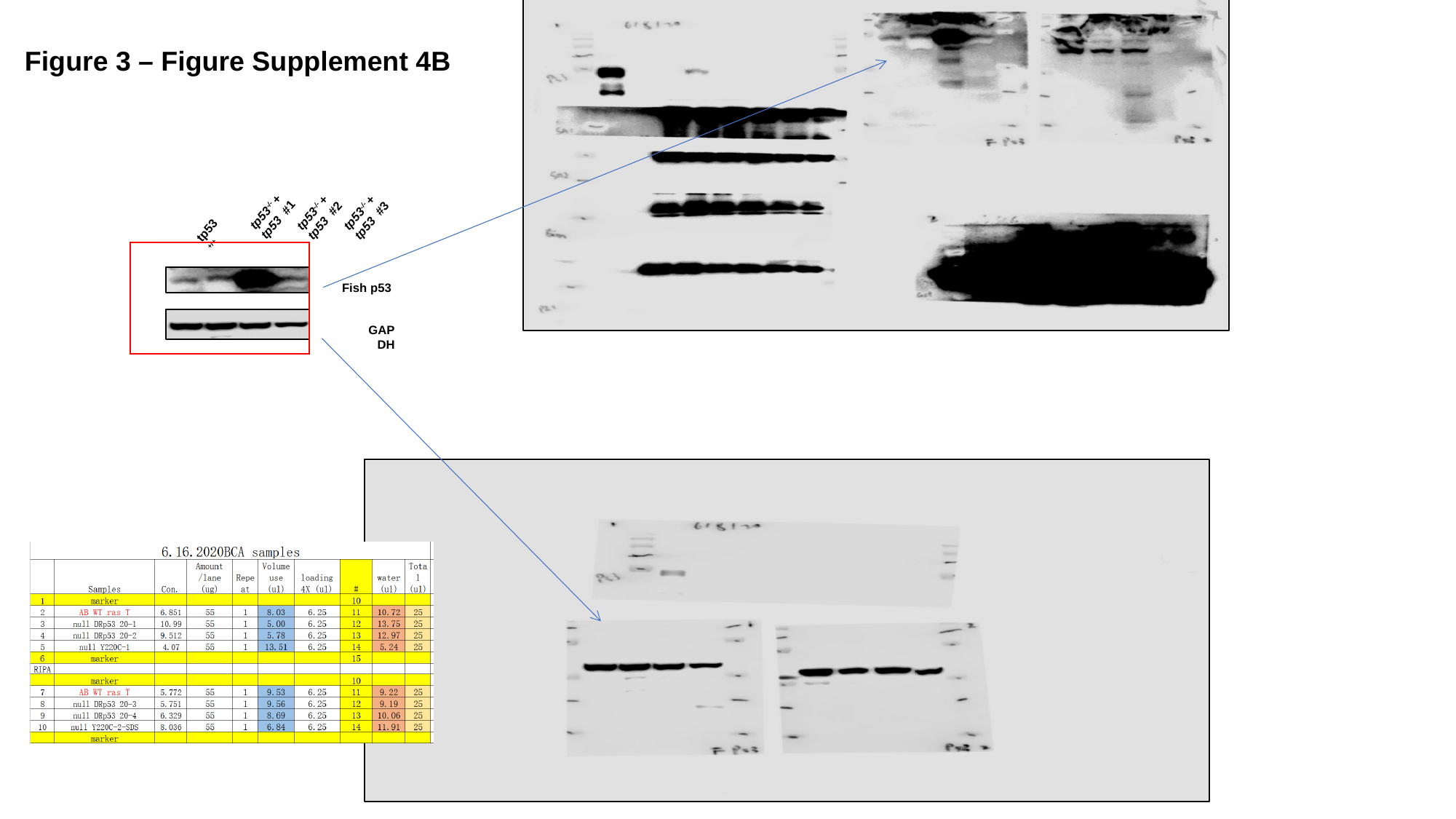

Figure 3 – Figure Supplement 4B
tp53-/- + tp53 #1
tp53-/- + tp53 #3
tp53-/- + tp53 #2
tp53+/+
Fish p53
GAPDH

## Slide 6
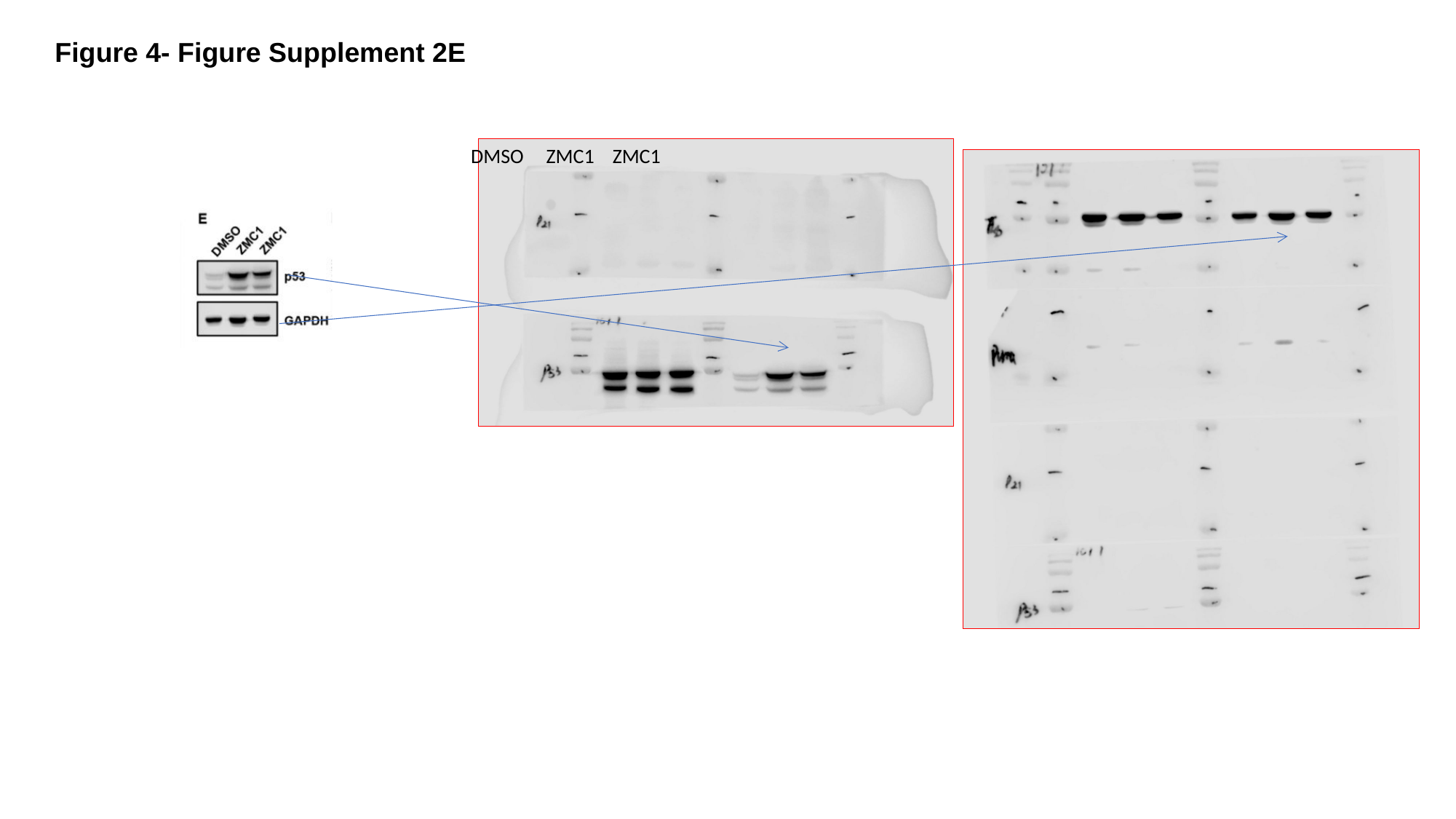

Figure 4- Figure Supplement 2E
DMSO ZMC1 ZMC1

## Slide 7
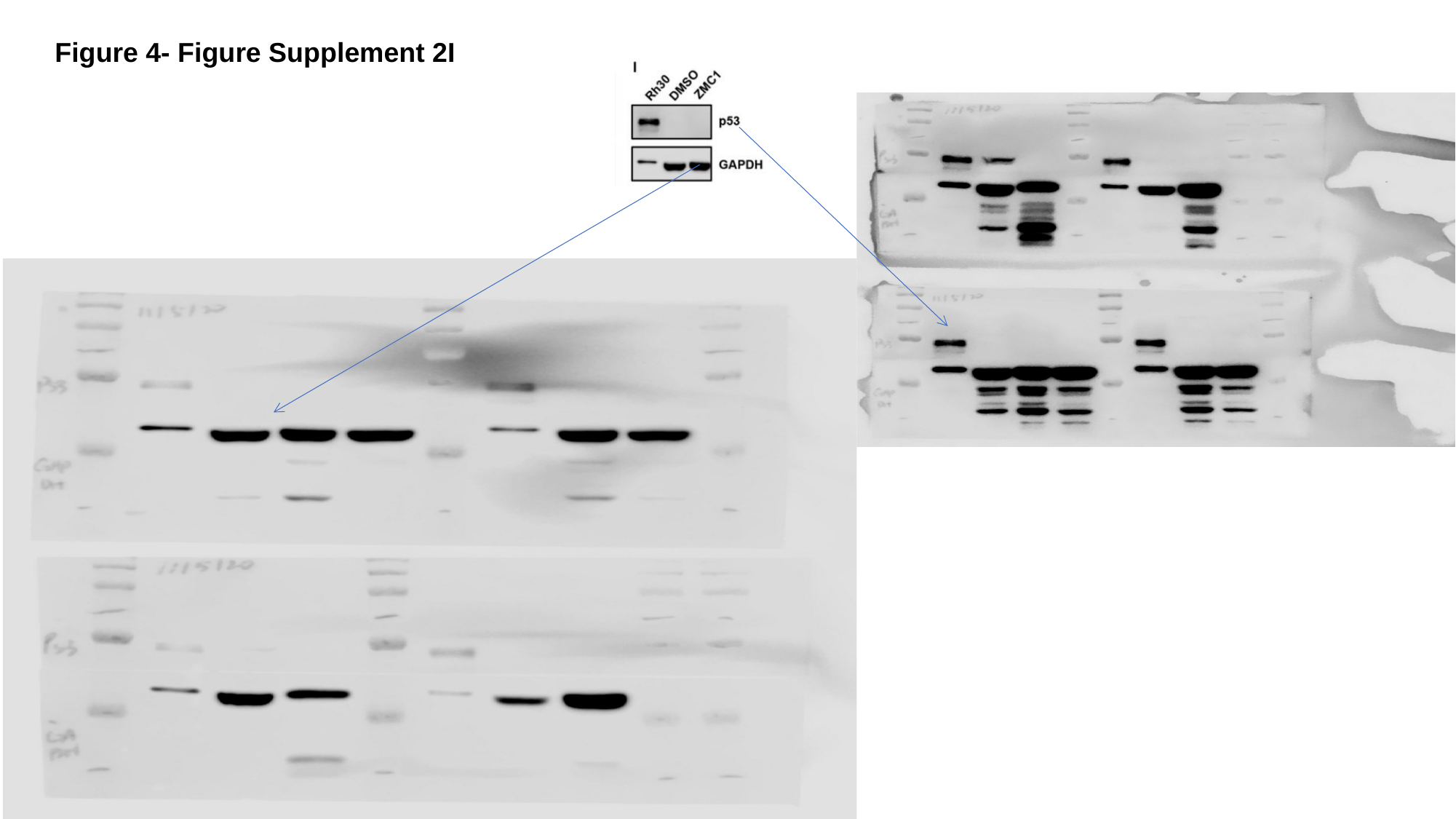

Figure 4- Figure Supplement 2I

## Slide 8
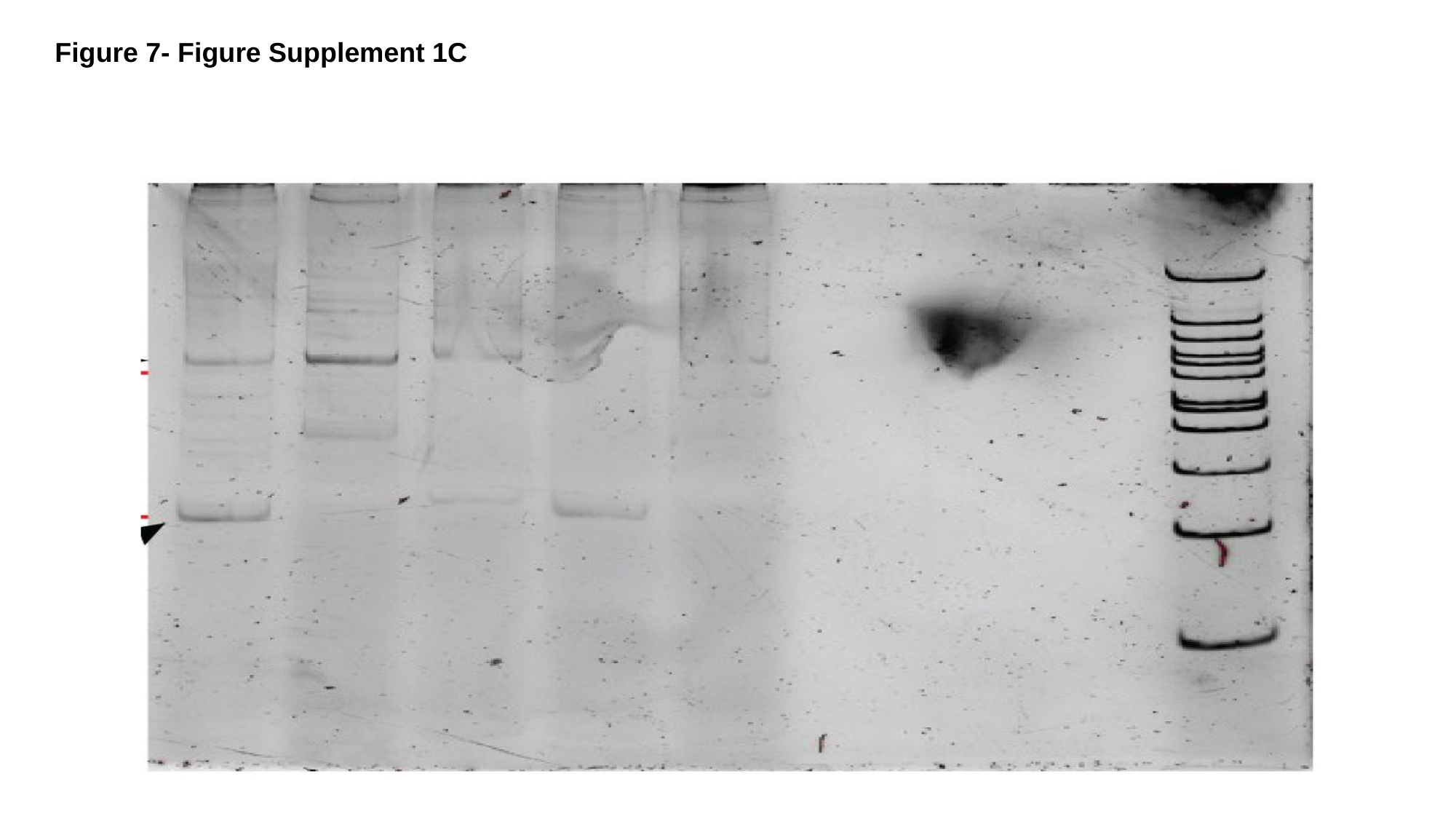

Figure 7- Figure Supplement 1C
